# Targeting Cancer-Specific Mutations with RNA-Triggered Chromatin Shredding

**DOI:** 10.1038/s41586-026-10738-7

**Published:** 2026-06-08

**Authors:** Jingkun Zeng, Zhiyuan Cheng, Huadong Chen, Zhaojun Wang, Jared Thompson, Kadin T. Crosby, Hesong Han, Arushi Singhal, Wayne Ngo, Chenglong Xia, Daniel Rosas-Rivera, Zeyuan Zhang, Min Hyung Kang, Ying Mao, Morgan E. Diolaiti, Giselle C. Lee, John F. X. Diffley, Yixuan Song, Longhui Qiu, Nathan M. Krah, Niren Murthy, Ryan N. Jackson, Yang Liu, Alan Ashworth, Jennifer A. Doudna

**Affiliations:** 1 Gladstone Institute of Data Science and Biotechnology, San Francisco, CA, USA; 2 Gladstone-UCSF Institute of Genomic Immunology, San Francisco, CA, USA; 3 Innovative Genomics Institute, University of California, Berkeley, Berkeley, CA, USA; 4 Department of Molecular and Cell Biology, University of California, Berkeley, Berkeley, CA, USA; 5 Howard Hughes Medical Institute, University of California, Berkeley, Berkeley, CA USA; 6 Molecular Biophysics and Integrated Bioimaging Division, Lawrence Berkeley National Laboratory, Berkeley, CA, USA; 7 California Institute for Quantitative Biosciences, University of California, Berkeley, Berkeley, CA, USA; 8 Li Ka Shing Center for Genomic Engineering, University of California, Berkeley, Berkeley, CA, USA; 9 Department of Chemistry, University of California, Berkeley, Berkeley, CA, USA; 10 Helen Diller Family Comprehensive Cancer Center, University of California, San Francisco, CA; 11 Department of Biochemistry, University of Utah School of Medicine, Salt Lake City, UT, USA.; 12 Department of Bioengineering, University of California Berkeley, Berkeley, CA, USA; 13 Department of Chemistry and Biochemistry, Utah State University, Logan, UT, USA.; 14 The Francis Crick Institute, London, UK; 15 Department of Medicine, UCSF, San Francisco, California, USA.; 16 Department of Internal Medicine, University of Utah, Salt Lake City, UT, USA.

## Abstract

Genetic mutations that drive cancer often occur in tumor suppressor proteins, including the p53 transcription factor which is altered in ~40–50% of cases^1,2^. However, current therapies fail to target most such mutations because the mutant proteins typically lack defined drug-binding pockets, and restoring the endogenous function has proven challenging. Here, we programmed CRISPR-Cas12a2, an RNA-guided nuclease with *trans*-nucleolytic cleavage activities^3,4^, to selectively kill cancer cells by targeting cancer-specific transcripts. This approach limits cell growth by inducing *trans* shredding of chromatin, triggering DNA damage responses and cell death. Unlike existing methods, RNA-guided Cas12a2 senses cellular RNA signatures, enabling precise targeting of undruggable mutations. Transcript-activated chromatin shredding provides a new approach to precision disease treatments for undruggable targets.

CRISPR-Cas12a2 is an RNA-guided nuclease that cleaves both RNA and DNA in *trans* upon target RNA recognition^3,4^. In bacteria, phage or other foreign transcripts trigger Cas12a2-guide RNA (gRNA) catalyzed depletion of cellular nucleic acids to induce cell dormancy or death. This abortive infection mechanism is thought to prevent phage propagation within the bacterial community by killing just those cells expressing a Cas12a2-guide RNA-detectable transcript. Similar abortive infection strategies are also employed by CRISPR-Cas13, an RNA-knockdown nuclease with *trans*-RNA cleavage activity^5–8^. While efforts in mammalian systems have largely focused on minimizing Cas13 *trans*-cleavage to improve RNA knockdown tools, we reasoned that *trans*-cleavage activities could instead be leveraged to selectively kill cells expressing particular mRNA sequences.

The field of cancer biology has long appreciated the utility of targeted cell killing because tumor cells express aberrant or mutated proteins not found in healthy tissue. *TP53,* encoding the most common cancer-associated transcription factor p53, is mutated in ~40–50% of all cancers and up to 70–90% of ovarian, NSCLC (non-small cell lung cancer) and pancreatic tumors^1,2,9^. *TP53* mutations also tend to be clonal, arising early and persisting within a heterogenous population of tumor cells^10,11^. Restoring p53 function for tumor regression has been considered the “holy grail” of cancer therapy^12–22^. However, no approved therapies are available to target the p53 protein due to its lack of druggable pockets and the difficulty of activating defective transcription factors. Furthermore, a potential side effect of p53 activators is that non-targeted p53 activation can induce senescence and whole-genome duplication^23–25^, causing significant dose-limiting toxicities^26–29^. One alternative approach would be just killing the cells expressing mutant proteins.

Here, we explored the potential for CRISPR-Cas12a2 to selectively target mammalian cells expressing specific mRNAs, including mutant *TP53* transcripts. We found that, upon RNA-guided RNA target recognition, Cas12a2 cleaves eukaryotic chromatin in *trans*, triggering DNA damage responses and cell death in mammalian cells (Fig. 1a). We show that mRNAs encoding mutant undruggable proteins can be targets for triggering Cas12a2-mediated killing. In particular, by utilizing gRNAs targeting single nucleotide variants (SNVs), we were able to selectively kill cells expressing several *TP53* point mutations. We show that this approach is highly specific, causing DNA damage-induced cell death only in the presence of the mutant but not the wild-type transcript. Delivering Cas12a2 mRNA and its guide RNA targeting c-MYC and p53 R248Q transcripts using lipid nanoparticles (LNPs) reduced tumor burden *in vivo* in mouse models. Together, these data suggest that cancer-specific cell targeting by transcript-activated chromatin shredding could be a useful new approach to cancer therapy.

## Results

### Cas12a2 shreds chromatin in trans

**Fig 1.**
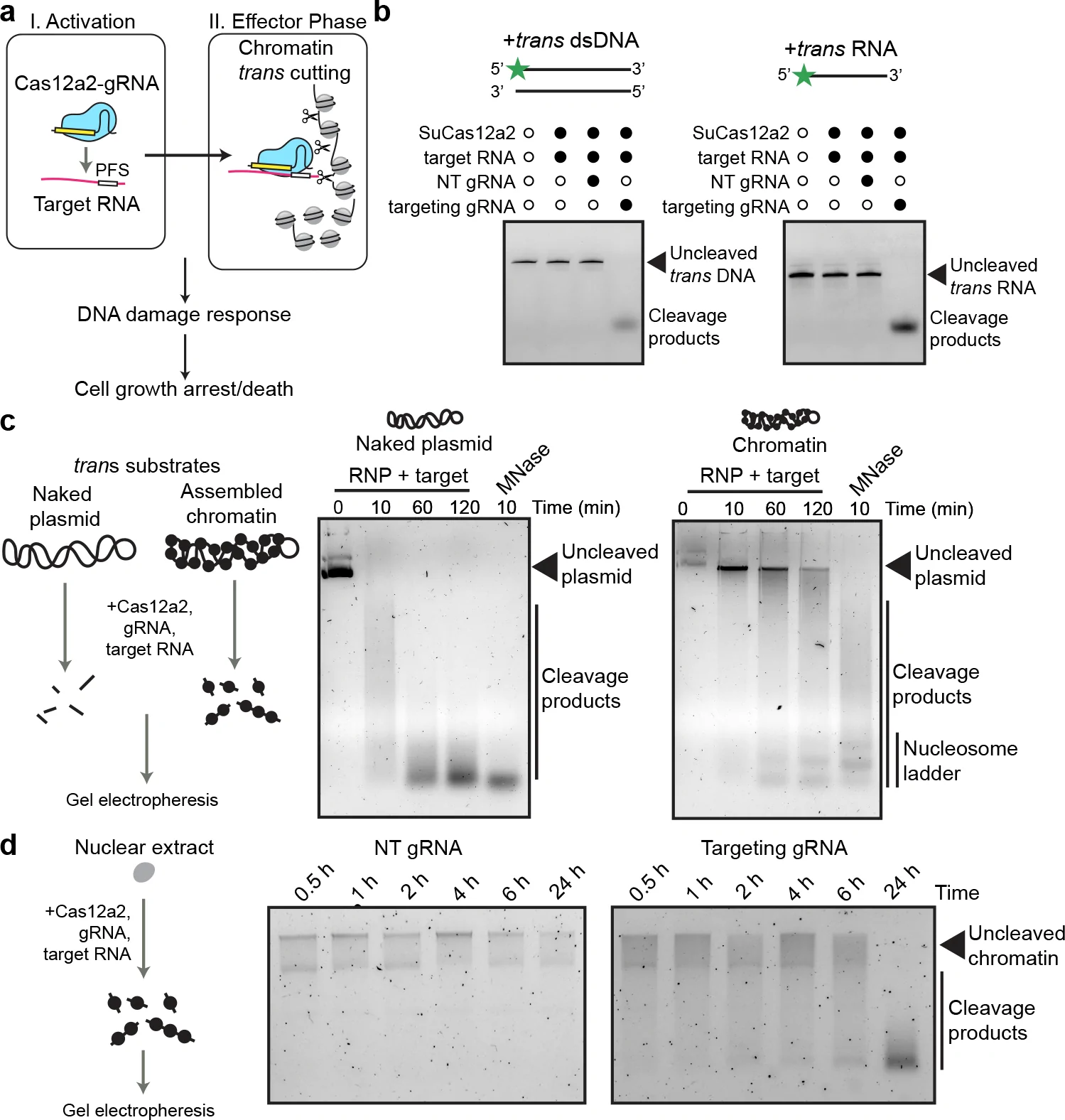
Biochemical analysis of chromatin *trans*-cleavage by Cas12a2. **a,** Schematic showing chromatin *trans*-cleavage and mammalian cell killing by Cas12a2 upon RNA targeting. PFS, protospacer flanking sequence. **b,** *Trans*-cleavage of FAM-labelled non-target ssRNA and dsDNA substrates by purified Cas12a2 protein after 2 h (n=3). NT, non-targeting. **c,** Time-course analysis of *trans*-cleavage of a naked supercoiled plasmid and a plasmid assembled into chromatin by Cas12a2-gRNA ribonucleoprotein (RNP) incubated with the target RNA (n=3). MNase, micrococcal nuclease. **d,** Time-course analysis of *trans*-cleavage of chromatin in HEK293 nuclear extracts by Cas12a2 RNPs (n=3). NT, non-targeting.

We first examined *trans* cleavage activity *in vitro* by Cas12a2 using purified SuCas12a2 ribonucleoproteins (RNPs) with fluorescently labelled linear nucleic acid substrates. Previous studies in bacteria identified a protospacer flanking site (PFS) requirement for SuCas12a2^3^, with a weak consensus 5-nt sequence of 5′-GAAAG-3′. However, adenine-rich motifs appear sufficient for activation, as one or two adenines within the middle three nucleotides of this 5-nt motif can support SuCas12a2 activity^3^. Accordingly, we designed gRNAs and target RNAs containing compatible PFS sequences. SuCas12a2 RNPs specific for the target RNA degraded both FAM-labelled RNA and dsDNA *trans* substrates (Fig. 1b). SuCas12a2 RNPs also cleaved supercoiled plasmid DNA *in trans* following RNA targeting (Fig. 1c), whereas control SuCas12a2 RNPs containing non-targeting guide RNAs did not induce RNA or DNA cleavage (Fig. 1b). These results are consistent with previous work showing that Cas12a2 degrades naked nucleic acids *in trans*^3,4^.

We next tested whether Cas12a2 RNPs can cleave chromatin, the context of nuclear DNA in eukaryotic cells. A 10.6 kb plasmid assembled into chromatin was gradually degraded *in trans* by SuCas12a2 RNPs upon RNA targeting, although at a slower rate than was observed for the naked plasmid (Fig. 1c). A distinct ladder of DNA cleavage products formed after 1 h of incubation with SuCas12a2 RNPs, corresponding to the sizes of mono-, di- and tri-nucleosomes, suggesting SuCas12a2 preferentially cleaved linker regions between nucleosomes. To test whether Cas12a2 RNPs can also degrade mammalian chromatin, we incubated nuclei extracted from HEK293 cells with SuCas12a2 RNPs, and a target RNA. Whereas RNPs containing a non-targeting (NT) gRNA induced no observable cleavage, RNPs containing a gRNA with sequence complementarity to target RNA led to degradation of HEK293 chromatin (Fig. 1d). These results suggest that Cas12a2 can serve as an RNA-guided chromatin shredder (Fig. 1a).

### Cas12a2 targeting kills mammalian cells

**Extended data fig 1.**
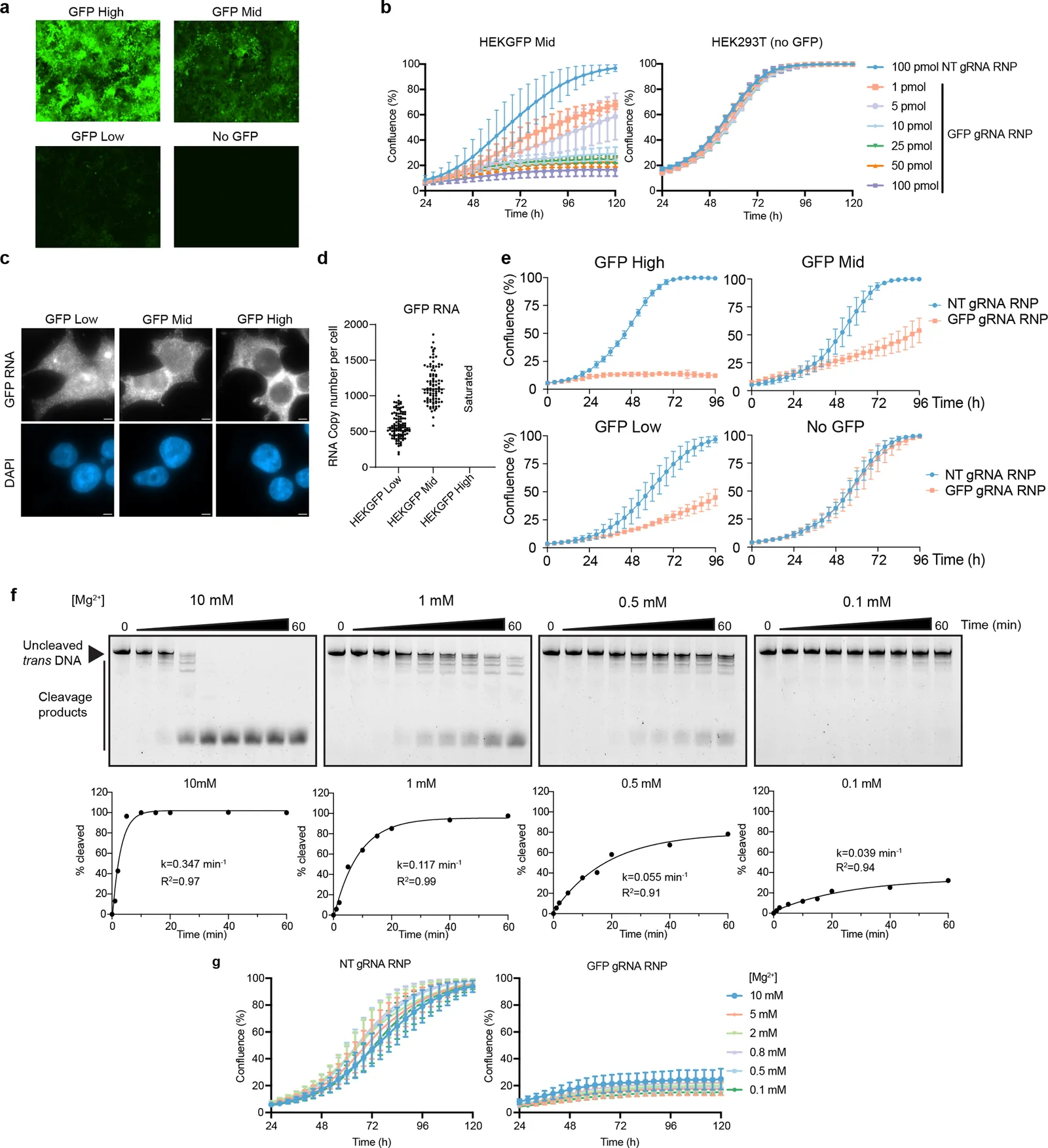
Cas12a2 activity in mammalian cells is influenced by target transcript abundance and Mg^2+^ concentration. **a,** Representative green channel images of HEK293 cells expressing different levels of GFP (High, Mid, Low) (n=2). **b,** Representative growth curves from three independent experiments of HEK293 cells (Mid or no GFP expression) nucleofected with different concentrations of Cas12a2-GFP gRNA RNP (mean ± SEM of three technical repeats is shown). **c,** Representative smFISH cell images. Scale bar, 5 μm. **d,** Quantification of RNA numbers from smFISH images. Bar indicates the median (n=109 for HEKGFP Low, n=94 for HEKGFP Mid). **e,** Representative growth curves from two independent experiments of HEKGFP cells in **b** nucleofected with 5pmol of GFP gRNA RNP (mean ± SEM of three technical repeats is shown). **f,** Time-course *in vitro* DNA trans-cleavage analysis by Cas12a2 in different concentrations of Mg^2+^ (n=2 independent experiments; k values are mean). **g,** Representative growth curves from three independent experiments of HEK293-GFP (mid) cells nucleofected with RNP pre-incubated in different concentrations of Mg^2+^ (mean ± SEM of three technical repeats is shown).

**Fig 2.**
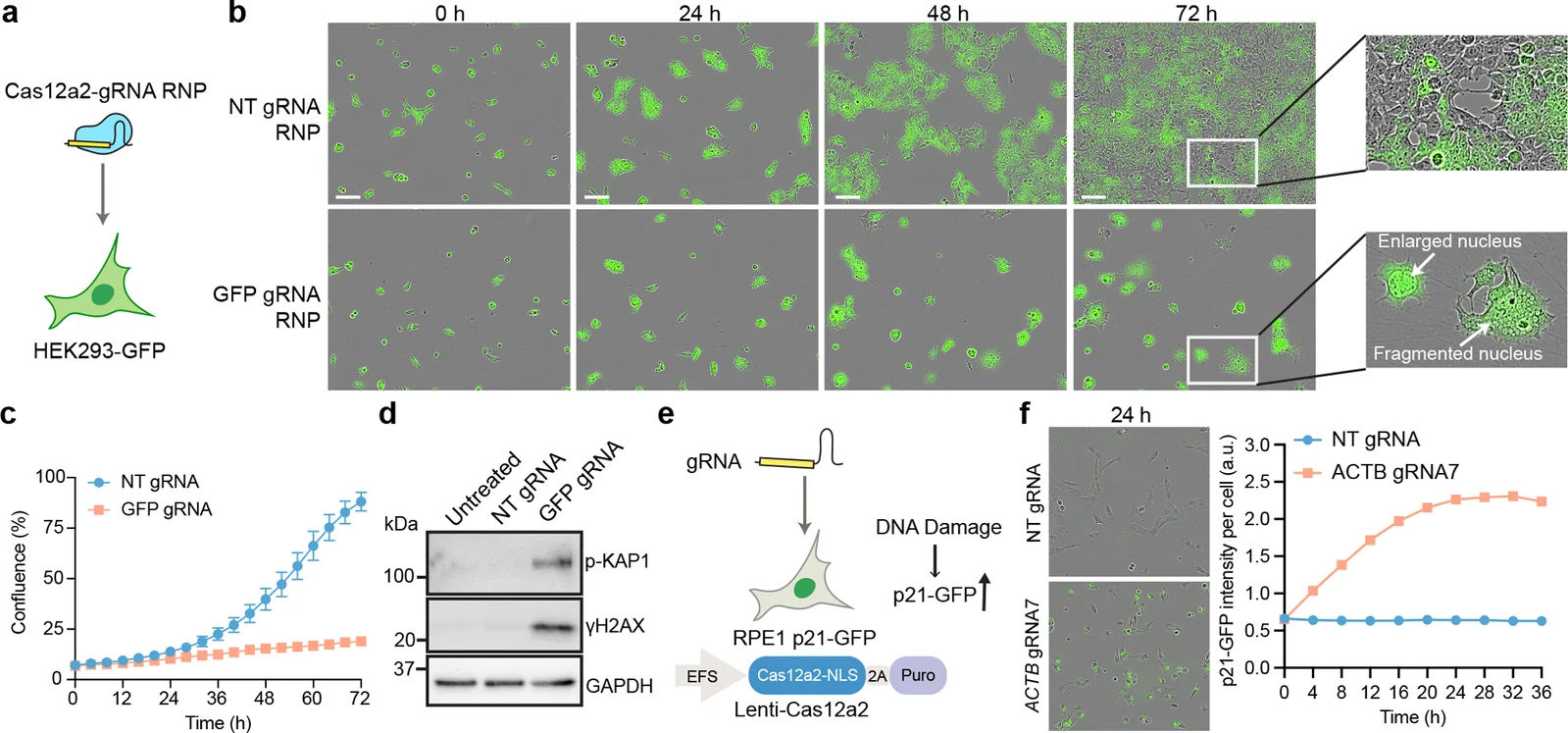
Cas12a2 induces acute DNA damage and cell cycle arrest upon RNA targeting in mammalian cells. **a,** Nucleofection of Cas12a2 RNP complexes into HEK293 cells expressing green fluorescent protein (GFP). **b,** Representative time-lapse images of HEK293-GFP cells nucleofected with Cas12a2 RNPs with the NT gRNA (NT gRNA RNP) or the GFP-targeting gRNA (GFP gRNA RNP). Merged green and phase contrast channels are shown. Scale bar, 100 μm. **c**, Cell confluence measurement of images taken every 4 h in **b** (n=4; mean ± SEM is shown)**. d,** Immunoblots showing expression of DNA damage markers in HEK293-GFP cells 48 h following RNP nucleofection (n=3). **e,** A genotoxic stress reporter RPE1 cell line expressing GFP-tagged DNA damage response factor p21, and transduced with Cas12a2-NLS under an EFS promoter. 2A: self-cleaving peptide. Puro: puromycin resistance gene. **f,** Time-course measurement of p21-GFP signal following transfection of an *ACTB*-targeting gRNA. Representative images at 24 h are shown.

RNA-guided chromatin shredding could potentially induce programmed killing of mammalian cells. To test Cas12a2 activation in mammalian cells, we nucleofected different doses of SuCas12a2 RNPs targeting GFP mRNA transcripts into HEK293T cells stably expressing green fluorescent protein (GFP) (hereafter HEKGFP) (Fig. 2a). Live cell imaging showed that almost no cell proliferation occurred in HEKGFP cells nucleofected with 10–100 pmol SuCas12a2 RNP containing GFP-complementary gRNA (GFP gRNA RNP), whereas control HEK293T cells not expressing GFP were unaffected (Fig. 2a–c; Extended Data Fig. 1a, b). The nucleofected HEKGFP cells contained enlarged or fragmented nuclei after 72 h, indicative of mitotic bypass or mitotic catastrophe after extensive DNA damage leading to a loss of cell viability^25^ (Fig. 2b). These cells also showed increased expression of γH2AX, a marker for DNA double-stranded breaks, and phospho-KAP1 (S824), a marker for heterochromatic DNA damage^30^ (Fig. 2d), consistent with Cas12a2-mediated chromatin *trans*-cleavage.

To investigate whether target transcript abundance affects Cas12a2-induced cell death, we established single cell clones of HEK293T cells expressing high, medium (corresponding to HEKGFP used above) and low levels of GFP (referred to as HEKGFP High, HEKGFP Mid and HEKGFP Low) (Extended Data Fig. 1a). RNA-seq analysis of HEKGFP Mid cells showed that GFP transcripts had a CPM (counts per million) of 678 (Supplementary Table 5), ranking 140th among all protein-coding transcripts in this cell line, indicating a highly expressed but physiologically relevant target. Using single-molecule fluorescence *in situ* hybridization (smFISH), we quantified GFP mRNA transcripts in these cells, detecting ~550 transcripts per cell in HEKGFP Low, ~1,100 in HEKGFP Mid, and an unquantifiable number in HEKGFP High due to image saturation (Extended Data Fig. 1c, d). Following nucleofection with a low dose (5 pmol) GFP gRNA RNP, HEKGFP High cells showed almost no cell proliferation, whereas HEKGFP Mid and HEKGFP Low showed milder growth defects (Extended Data Fig. 1e). These results suggest that the extent of Cas12a2-mediated cell death in mammalian cells correlates with target transcript abundance.

### Cas12a2 activity depends on Mg^2+^

Previous biochemical assays using Cas12a2 RNPs were performed at 10 mM MgCl_2_^3,4^, whereas free magnesium ion concentrations in mammalian cells are ~0.1–1 mM^31^. CRISPR-Cas9 enzyme activity is known to be lower at lower Mg^2+^ levels^32^, so we asked whether Cas12a2 is similarly affected by Mg^2+^ concentration. *In vitro trans* cleavage assays over a range of Mg^2+^ concentrations showed reduced activity at lower Mg^2+^ concentrations, but detectable *trans* DNA cleavage was observed even at 0.1 mM Mg^2+^ (Extended Data Fig. 1f).

To test whether Mg^2+^ concentration affects RNP formation or cleavage activity, we pre-incubated SuCas12a2 protein with GFP gRNA at Mg^2+^ concentrations ranging from 0.1 mM to 10 mM, followed by nucleofection into HEK293-GFP cells. Regardless of the Mg^2+^ concentration used for RNP pre-incubation, cells were induced to stop growing with the same efficiency (Extended Data Fig. 1g), indicating that Cas12a2 RNP activity remains sufficient to induce mammalian cell death at physiological Mg^2+^ levels.

### Cas12a2 triggers acute DNA damage

HEK293T cells express SV40 large T antigen which inhibits key cell cycle and DNA repair regulators RB (retinoblastoma) and p53^33,34^. To examine Cas12a2-induced stress responses in a more normal mammalian context, we used hTERT-RPE1 (hereafter RPE1), a non-transformed epithelial cell line immortalized by telomerase and bearing wild-type p53 activity. We stably expressed nucleoplasmin nuclear localization signal (NLS)-tagged SuCas12a2 in RPE1 cells under an EF1α short (EFS) promoter, and recombined GFP into the C-terminus of the p53 downstream cell cycle inhibitor *CDKN1A* (encoding p21) (Fig. 2e). This created a genotoxic stress reporter cell line, wherein GFP expression increases in response to DNA damage as a result of elevated p21 expression.

**Extended data fig 2.**
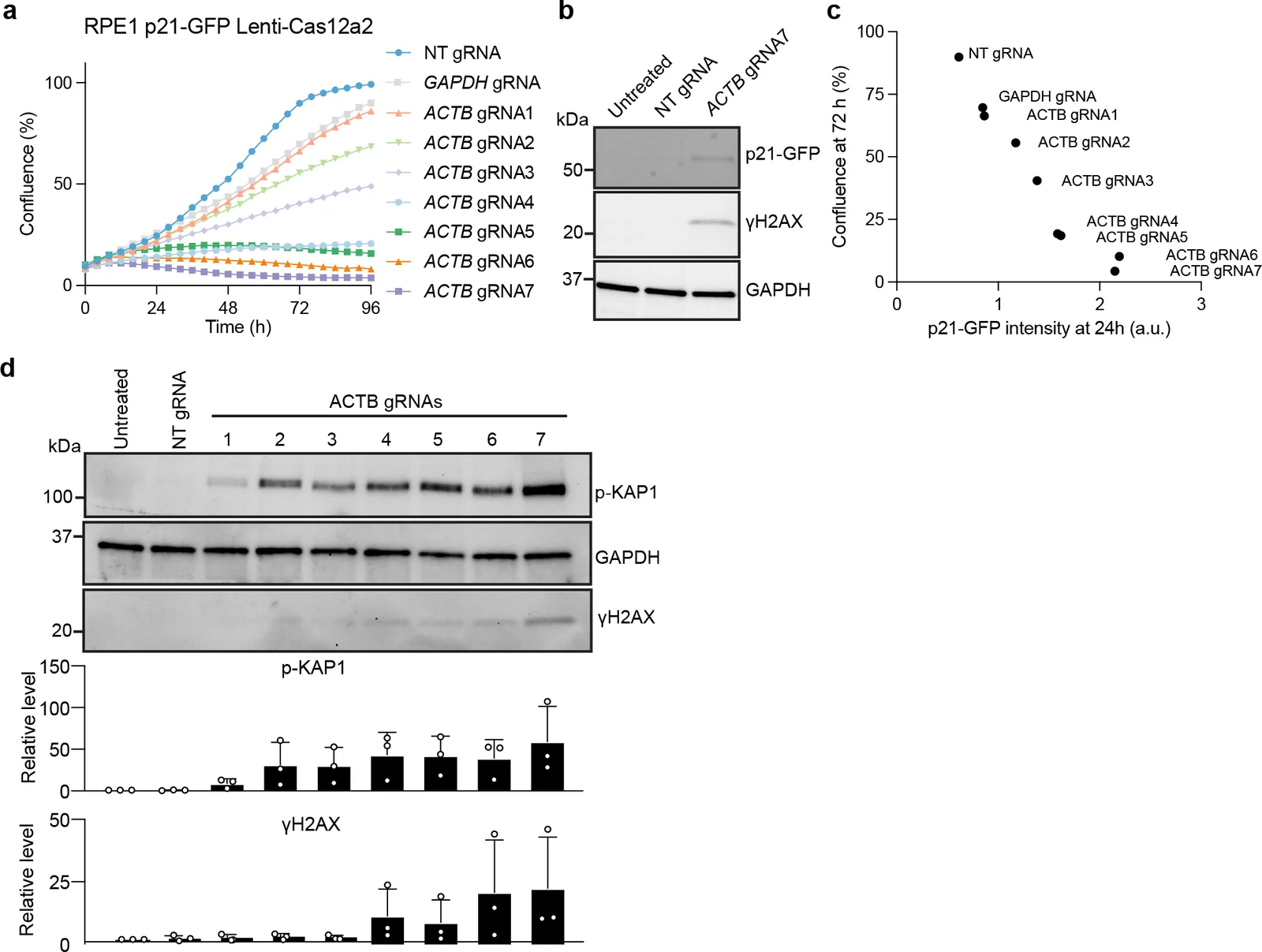
Targeting endogenous transcripts induces DNA damage and growth inhibition. **a,** Averaged growth curves of RPE1 p21-GFP Lenti-Cas12a2 cells transfected with 38.4 nM gRNAs targeting *ACTB* or *GAPDH.* (n=2 independent experiments; mean is shown). **b,** Immunoblots showing expression of DNA damage markers in RPE1 p21-GFP LentiCas12a2 cells 48 h following transfecting 38.4 nM *ACTB* gRNA7 (n=3). **c,** Correlation of cell confluence of RPE1 p21-GFP LentiCas12a2 cells in **a** with p21-GFP intensity (n=2 independent experiments; mean is shown). **d,** Immunoblots showing expression of DNA damage markers of cells in **a.** Normalized expression values against GAPDH are shown in the bar graph as mean ± SD (n=3 independent experiments).

Using this reporter cell line, we assessed stress responses upon Cas12a2 activation. Transfecting gRNAs targeting *ACTB* (encoding β-ACTIN) or *GAPDH* transcripts led to attenuated cell growth and expression of DNA damage markers (Extended Data Fig. 2a, b). Among the gRNAs tested, the ones that produced the strongest cell growth inhibition also caused the largest increase in GFP signal and expression of DNA damage markers (Extended Data Fig. 2c, d), consistent with DNA damage-induced growth arrest. GFP expression increased within 4 hours after transfecting *ACTB* gRNAs, peaking at around 24–36 h (Fig. 2f), suggesting Cas12a2 cleaved chromatin in mammalian cells shortly after target transcript recognition to trigger DNA damage responses.

### Targeting over-expressed oncogenes

**Extended data fig 3.**
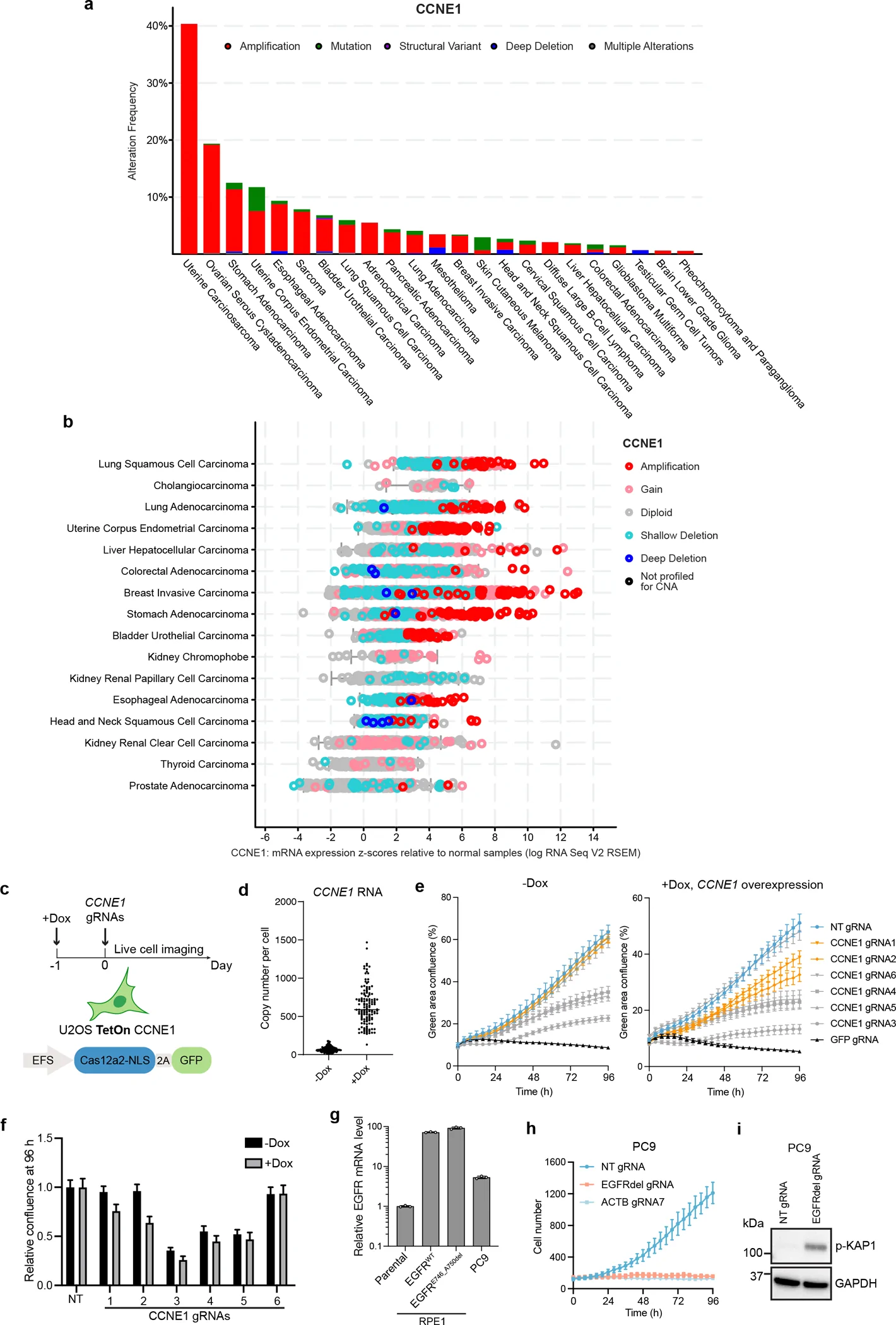
Cas12a2 targeting of CCNE1 and mutant EGFR transcripts inhibits cancer cell growth. **a,** Prevalence of *CCNE1* alterations in different cancers from TCGA Pan-Cancer Atlas studies. **b,** *CCNE1* mRNA expression levels in different cancers from TCGA Pan-Cancer Atlas studies. **c,** Testing of the *CCNE1*-targeting gRNAs in U2OS cells expressing CCNE1 under a Dox-inducible promoter and stably expressing Cas12a2. **d,** Quantification of smFISH measurement of RNA numbers. Bar indicates the median (n=148 cells for -Dox, n=126 for +Dox). **e,** Representative growth curves from two independent experiments of U2OS cells in **c (**mean ± SEM of sixteen technical repeats is shown). **f,** Quantification of cell number normalized against NT gRNA treated cells in **e (**mean ± SEM is shown)**. g,** RT-qPCR analysis of EGFR RNA levels in indicated cells (n=3 technical replicates; mean ± SEM is shown). **h,** Representative growth curves from three independent experiments of Cas12a2-integrated PC9 cells following transfection of indicated gRNAs at 38.4 nM (mean ± SEM of three technical repeats is shown). **i,** Immunoblots showing expression of DNA damage markers in PC9 cells 48 h following Cas12a2 targeting of the EGFR E746_A750del mutant transcript (n=3).

Elevated expression of oncogenes is a common driver of tumorigenesis. For example, *CCNE1* (encoding cyclin E1) and *MYC* are frequently amplified in various cancers^35,36^, leading to high transcript levels (Extended Data Fig. 3a, b). Since higher target expression levels correlate with more efficient Cas12a2 RNP-induced cell killing (Extended Data Fig. 1c–e), we reasoned that Cas12a2 RNPs might selectively kill cancer cells expressing high levels of oncogenes. To explore this, we used U2OS cells expressing doxycycline (Dox)-inducible cyclin E1 (hereafter U2OS TetOn CCNE1) (Extended Data Fig. 3c), which can be induced to over-express cyclin E1 at a level comparable to patient-derived cancer cells^25,37^. Before and after Dox treatment, there were ~70 and ~640 *CCNE1* transcripts per cell respectively (Extended Data Fig. 3d). We tested six gRNAs targeting the *CCNE1* mRNA transcript in U2OS TetOn CCNE1 cells stably expressing Cas12a2. Whereas some gRNAs (gRNA 3, 4 and 5) induced cell growth defects in both untreated cells and Dox-treated cells, gRNA 1 and 2 induced growth defects selectively in Dox-treated cells (Extended Data Fig. 3e, f). These observations suggested that a targeting window exists to distinguish between high and low levels of the same transcript. Taken together, these results imply that transcripts expressed at elevated levels in cancer cells can be targeted for Cas12a2-mediated cell killing.

### Targeting cancer-specific neo-junctions

**Fig 3.**
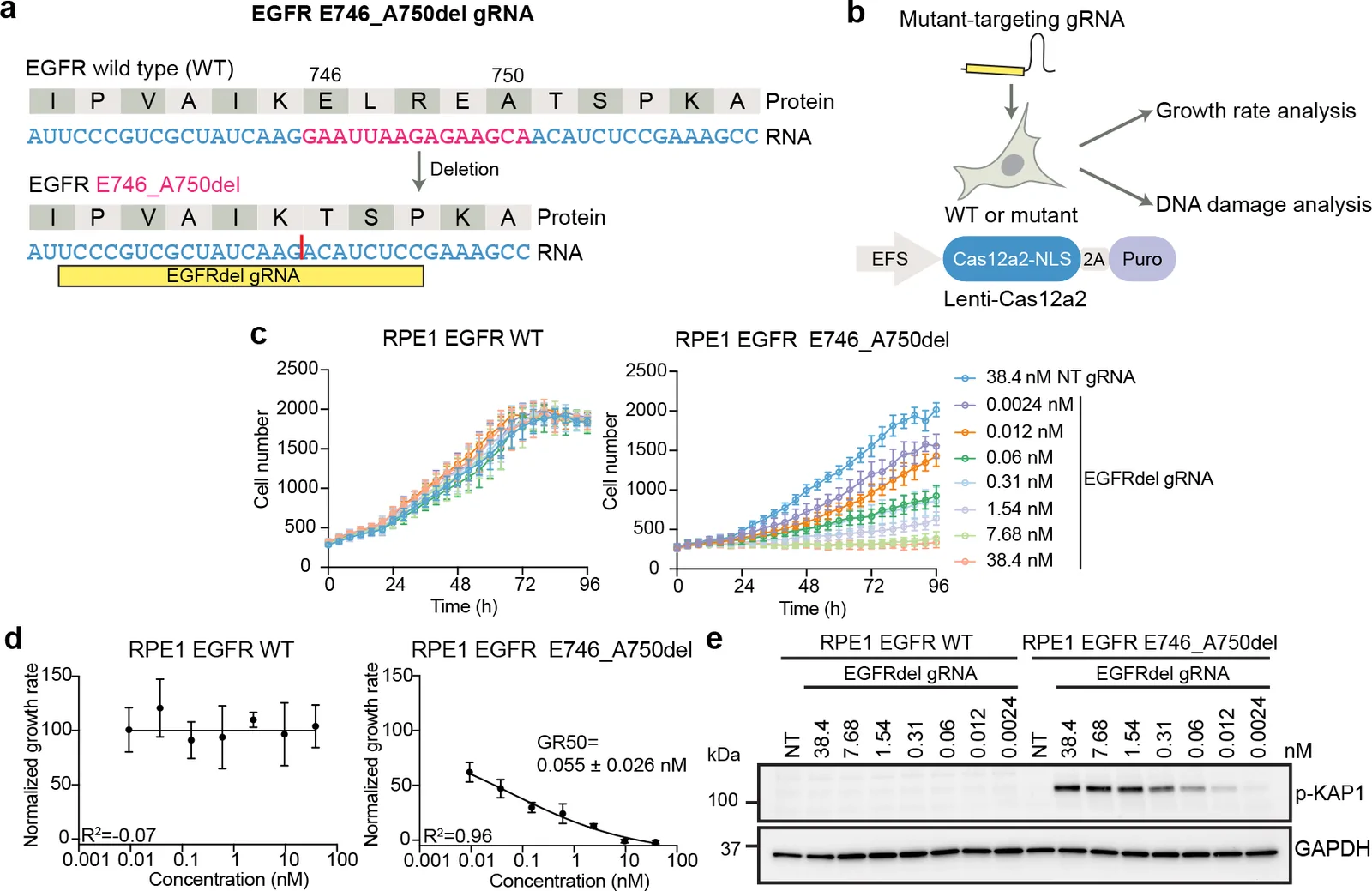
Selective killing of cells harboring an *EGFR* in-frame deletion mutation. **a,** Design of a gRNA (EGFRdel gRNA) targeting the EGFR E746_A750del mutant transcript. **b,** Testing of the EGFRdel gRNA in cells expressing wild-type (WT) or mutant transcripts. These cells also stably express Cas12a2. **c**, Dose-response growth curves of RPE1 EGFR WT and RPE1 EGFR E746_A750del cells following transfection of the EGFRdel gRNA (mean ± SEM from three independent experiments is shown). **d**, GR50 (50% growth-rate inhibition concentration) analysis of the data shown in **c**. GR50 values are shown as mean ± SEM (n=3 independent experiments). **e,** Immunoblots showing expression of DNA damage markers in cells 24 h following Cas12a2 targeting of the EGFR E746_A750del mutant transcript (n=3).

In-frame insertion and deletion (indel) mutations can lead to hyperactivation of oncogenes. *EGFR* (epidermal growth factor receptor) exon 19 deletion mutations are common activating mutations found in NSCLC, making up ~45% of all *EGFR* mutations^38,39^. One frequent mutation is EGFR E746_A750 deletion (E746_A750del), which results from a 15-bp genomic deletion and produces a mutant transcript containing a unique deletion junction sequence (Fig. 3a). We reasoned that such mutation-specific junction sequences could be selectively targeted by Cas12a2 for mutation-dependent cell killing. To test this, we established RPE1 cells expressing EGFR E746_A750del (RPE1 EGFR E746_A750del) and EGFR WT (RPE1 EGFR WT) under an EF1α promoter using lentiviral transduction. Real-time quantitative PCR (RT-qPCR) confirmed comparable expression levels of mutant and wild-type EGFR transcripts in the two RPE1 cell lines (Extended Data Fig. 3g). We designed a Cas12a2 gRNA that is complementary to the deletion junction (EGFRdel gRNA), which is expected to anneal only to the mutant transcript and not to the wild-type transcript (Fig. 3a). RPE1 EGFR E746_A750del and RPE1 EGFR WT cells stably expressing Cas12a2 were transfected with titrations of EGFRdel gRNA and monitored for growth inhibition (Fig. 3b–d). EGFRdel gRNA induced robust growth inhibition in RPE1 EGFR E746_A750del cells, showing a GR50 (50% growth rate inhibition concentration) of 0.055 ± 0.026 nM, together with increased expression of DNA damage markers, whereas no detectable growth inhibition or DNA damage was observed in RPE1 EGFR WT cells under the same conditions (Fig. 3d, e). In addition, we tested the EGFRdel gRNA in an NSCLC cell line PC9 containing an endogenous EGFR E746_A750 mutation. The EGFRdel gRNA induced strong growth inhibition and expression of DNA damage markers (Extended Data Fig. 3h, i). This finding suggested that indel junctions in oncogenic transcripts can be targeted by Cas12a2 for selective cell killing.

### Targeting TP53 mutations

**Extended data fig 4.**
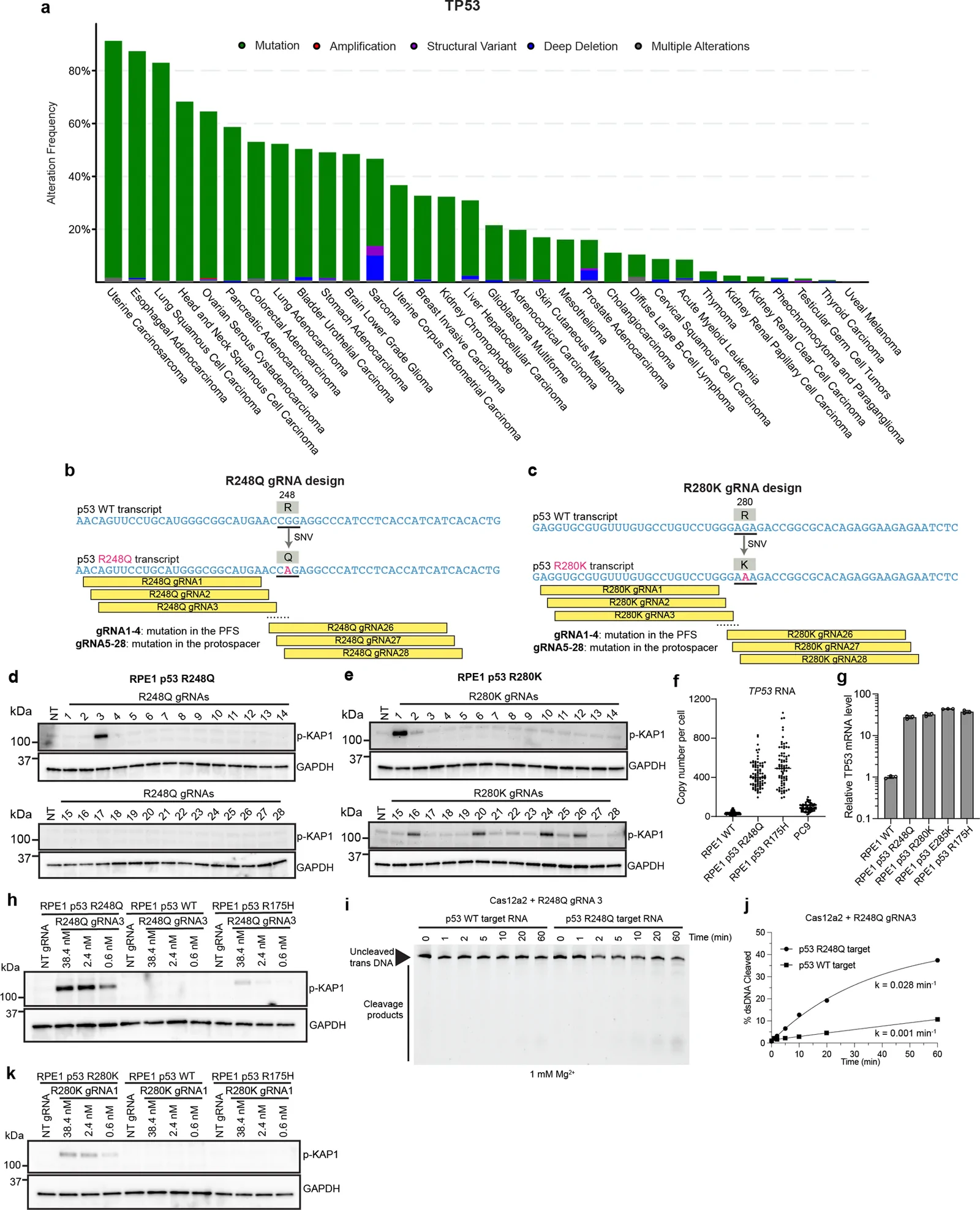
Cas12a2 selectively targets TP53 R248Q and R280K mutant transcripts. **a,** Prevalence of *TP53* alterations in different cancers from TCGA Pan-Cancer Atlas studies. **b and c,** Schematic showing annealing positions of screened gRNAs for targeting p53 R248Q and p53 R280K mutant transcripts. **d and e,** Immunoblots probing expression of DNA damage marker phospho-KAP1 24 h following transfection of screened gRNAs at 38.4 nM into Cas12a2-integrated RPE1 p53 R248Q and R280K cells (n=3). **f,** Quantification of RNA levels using smFISH. Bar indicates the median (n=96 for RPE1, n=88 for RPE1 p53 R248Q, n=79 for RPE1 p53 R175H, n=67 for PC9). **g,** Quantification of RNA levels using RT-qPCR (n=3 technical replicates; mean ± SEM is shown). **h,** Immunoblots probing expression of DNA damage marker phospho-KAP1 24 h following Cas12a2 targeting of p53 R248Q mutant transcripts with 38.4 nM gRNA in p53 target mutant cells, p53 WT cells and p53 R175H control cells (n=3). **i-j**, Time-course analysis of *in vitro trans*-cleavage of FAM-labelled dsDNA by Cas12a2 with R248Q gRNA3 in the presence of p53 R248Q RNA fragment or p53 WT RNA fragment. Quantification is shown in **j** (n=2 independent experiments; k values are mean)**. k,** Immunoblots probing expression of DNA damage marker phospho-KAP1 24 h following Cas12a2 targeting of p53 R280K mutant transcripts with 38.4 nM gRNA in p53 target mutant cells, p53 WT cells and p53 R175H control cells (n=3).

**Fig 4.**
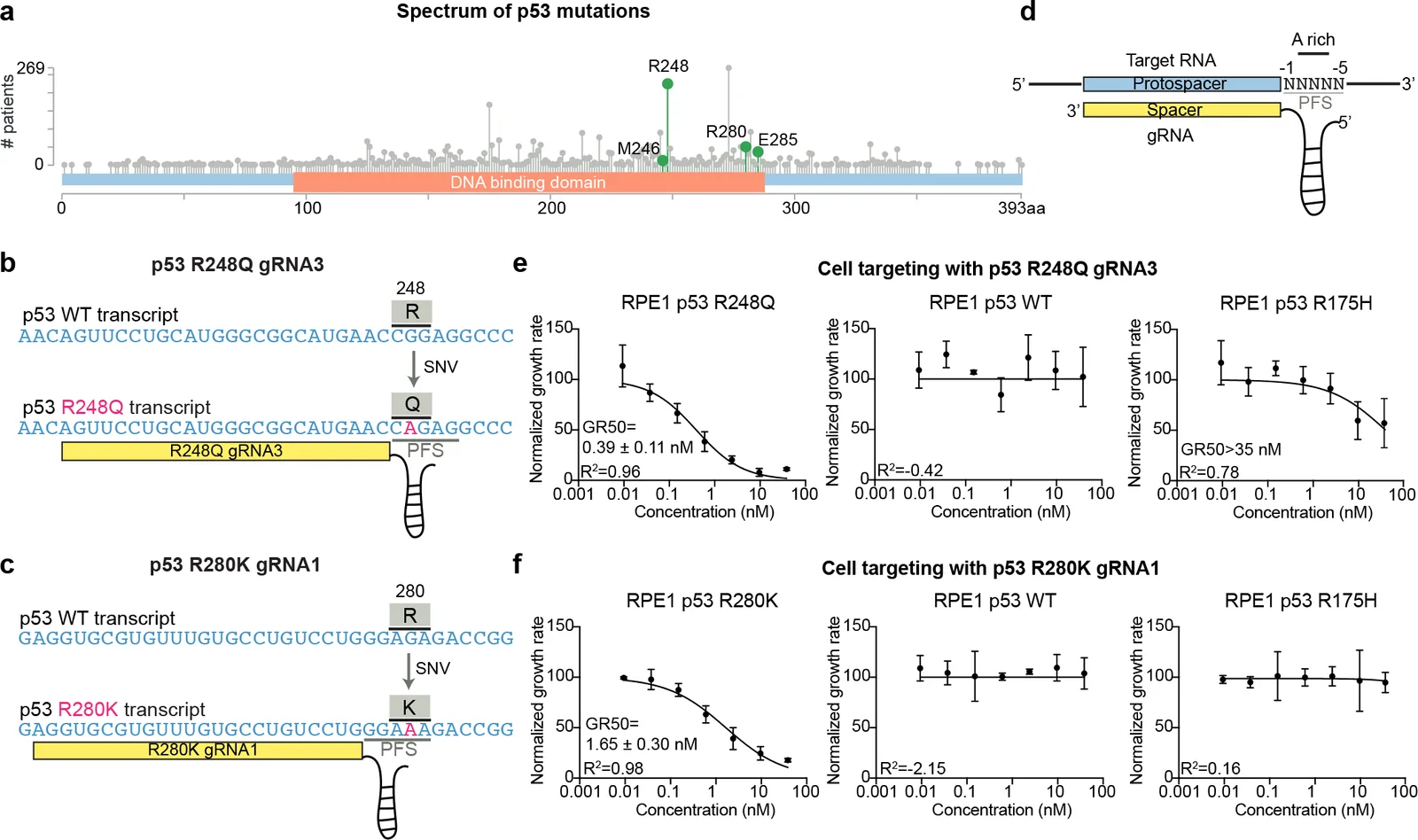
Selective killing of cells harboring *TP53* SNVs. **a**, Mutational spectrum of the p53 protein in tumor samples from 3,949 cancer patients from TCGA Pan-Cancer Atlas studies in the cBioPortal database. M246, R248, R280 and E285 residues targeted in this study are highlighted. **b and c**, Schematic showing R248Q gRNA3 and R280K gRNA1 targeting p53 R248Q and p53 R280K transcripts respectively. **d,** Schematic showing the protospacer flanking site (PFS) for Cas12a2 targeting. **e and f,** GR50 analysis of R248Q gRNA3 and R280K gRNA1 in Cas12a2-integrated cells expressing the target p53 mutation, p53 WT or the control mutant p53 R175H. GR50 values are shown as mean ± SEM (n=3 independent experiments).

Mutations in the p53 tumor suppressor protein, the most common driver for tumorigenesis (Extended Data Fig. 4a), often result from SNVs in the *TP53* gene sequence. Although mutations occur across the protein^40^, several ‘hotspot’ mutations are particularly common^41^ (Fig. 4a). For example, R248Q, caused by a G-to-A substitution in the gene sequence, makes up ~7% of all *TP53* mutations. We asked whether Cas12a2 can distinguish such SNVs in *TP53* mutant transcripts.

We generated RPE1 cells expressing the hotspot mutation p53 R248Q or another mutation, R280K, under an EF1α promoter, both of which introduce a G-to-A substitution in the transcript relative to wild type (Fig. 4b, c). To identify selective guides, we designed 28 gRNAs tiled across each mutation site, including guides targeting the mutation through the protospacer flanking site (PFS) and guides targeting the mutation within the protospacer (Extended Data Fig. 4b, c). We screened these guides by transfecting them into Cas12a2-expressing mutant cells and measuring induction of DNA damage markers (Extended Data Fig. 4d, e). Among the tested guides, one gRNA induced DNA damage in R248Q cells (R248Q gRNA3), whereas six gRNAs were active in R280K cells. Notably, the two strongest guides (R248Q gRNA3 and R280K gRNA1) both positioned the mutant adenine within PFS (Fig. 4b–d). Since Cas12a2 uses an adenine-rich PFS (Fig. 4d), the mutant adenine may enhance PFS recognition. Similar use of mutation-dependent recognition motifs has been seen with other CRISPR systems, such as allele-specific targeting by exploiting mutations that create or disrupt PAM sequences for Cas9^42,43^.

**Extended data fig 5.**
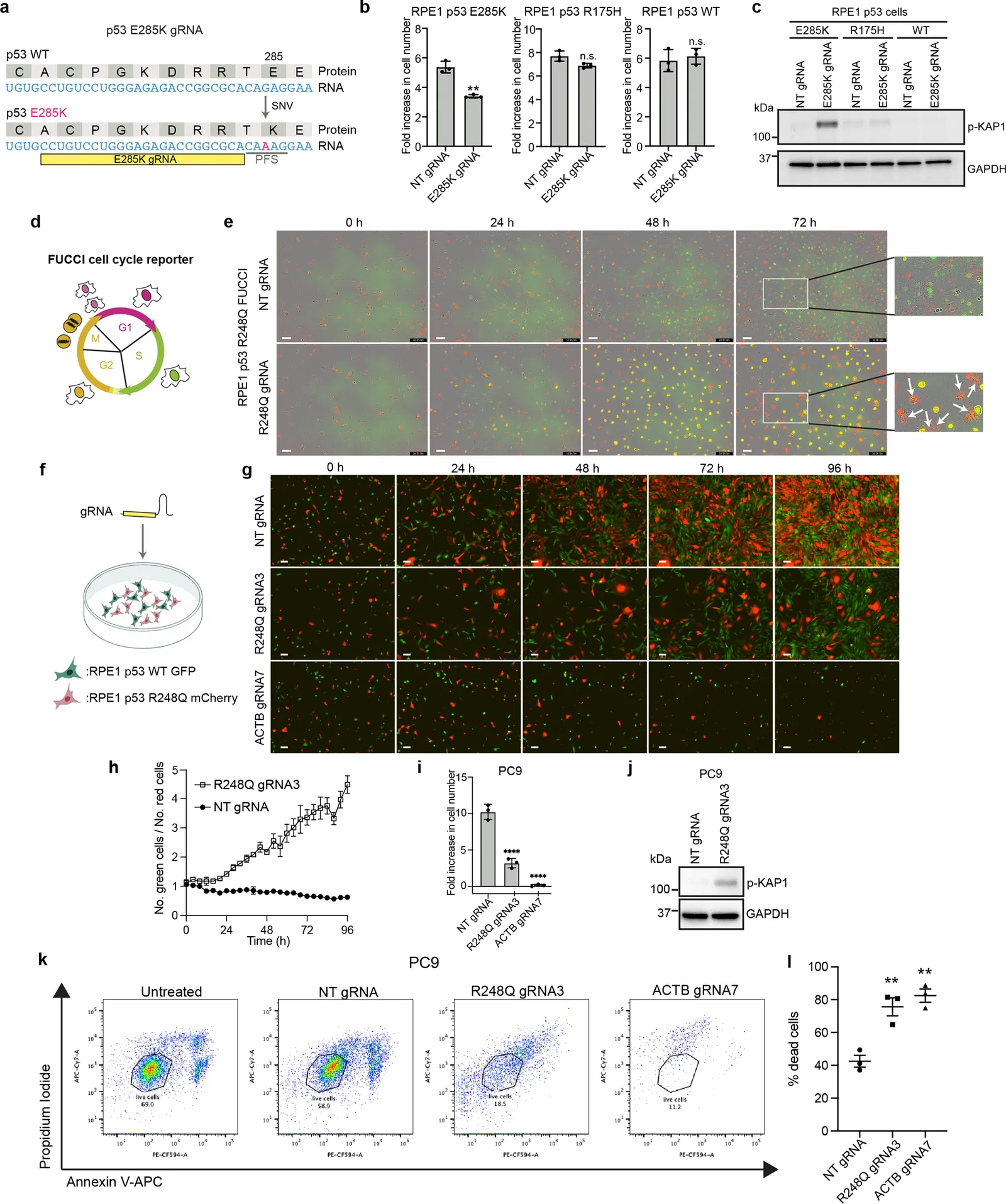
Cas12a2 shows selectivity when targeting mutations in the PFS. **a**, Schematic showing design of the p53 E285K gRNA. **b,** Fold‑increase in cell number over 96 h following Cas12a2 targeting of p53 E285K mutant transcript with 38.4 nM gRNA in target and control cells (n=3 independent experiments; mean ± SD is shown). Statistics: **p=0.0090; ns, non-significant, two-tailed unpaired t-test. **c**, Immunoblots showing expression of DNA damage marker phospho-KAP1 24 h following Cas12a2 targeting of the p53 E285K mutant transcript with 38.4 nM gRNA in target and control cells (n=3). **d,** Schematic of the FUCCI cell cycle reporter. **e,** Representative time-lapse images of RPE1 p53 R248Q cells expressing the FUCCI reporter following Cas12a2 targeting of the p53 R248Q mutant transcript with 38.4 nM R248Q gRNA3 (n=3). Merged green, red and phase contrast channels are shown. White arrows indicate cells with fragmented nuclei. Scale bar, 100 μm. **f-h**, Growth competition assay between RPE1 WT and R248Q cells, both stably expressing Cas12a2. Representative images of merged red and green channels are shown in **g**. Scale bar, 100 μm. Representative green to red cell ratios from three independent experiments are shown in **h** (mean ± SEM from three technical repeats is shown). **i**, Fold‑increase in cell number over 96 h following Cas12a2 targeting of the p53 R248Q mutant transcript with 38.4 nM gRNA in PC9 cells (n=3 independent experiments). Statistics: ****p<0.0001, one-way ANOVA with Dunnett’s test **j**, Immunoblots showing expression of DNA damage marker phospho-KAP1 48 h following Cas12a2 targeting of the p53 R248Q mutant transcript with 38.4 nM gRNA in PC9 cells (n=3; mean ± SD is shown). **k,** FACS gating of dead cell populations in PC9 cells 96 h following Cas12a2 targeting in **i**. **l,** Quantification of dead cell populations in PC9 cells 96 h following Cas12a2 targeting in **i** (n=3 independent experiments; mean ± SEM is shown). Statistics: **p=0.0033 for NT vs R248Q gRNA3, 0.0013 for NT vs ACTB gRNA7; ns, non-significant, one-way ANOVA with Dunnett’s test.

We next quantified the selectivity of R248Q gRNA3 and R280K gRNA1 by dose-response experiments in target mutant cells, wild-type (WT) RPE1 cells, and a control mutant line expressing p53 R175H at similar levels (~400–500 transcripts per cell) from the same EF1α promoter (Extended Data Fig. 4f). Both gRNAs showed strong selectivity for target mutant cells. R248Q gRNA3 induced robust growth arrest and DNA damage in p53 R248Q cells without affecting growth of WT cells (Fig. 4e; Extended Data Fig. 4g). At high concentrations, mild growth inhibition and DNA damage were observed in p53 R175H cells; however, the GR50 for R248Q cells (0.39 ± 0.11 nM) was ~100-fold lower than for R175H cells (>35 nM). Additionally, in biochemical assays, *trans* DNA cleavage by the R248Q gRNA3 RNP was ~28-fold faster in the presence of mutant target RNA than with WT RNA (Extended Data Fig. 4i, j). R280K gRNA1 showed excellent selectivity, potently inhibiting growth of R280K cells (GR50 = 1.65 ± 0.30 nM) while producing no detectable growth inhibition or DNA damage in WT or R175H cells at all concentrations tested (Fig. 4f; Extended Data Fig. 4h). Using the same design principle, we designed a gRNA targeting the p53 E285K mutation, which also introduces a G-to-A substitution in the transcript. This gRNA, which places the mutant adenine within the PFS, selectively induced DNA damage and growth inhibition in E285K cells, but not WT or p53 R175H control cells (Extended Data Fig. 5a–c). These results indicate that SNVs that create an activating PFS enable highly selective Cas12a2 targeting of mutant cells.

We observed by FUCCI (fluorescent ubiquitination-based cell-cycle indicator) live cell imaging^44^ that Cas12a2 targeting with R248Q gRNA3 caused extended G2 arrest in RPE1 p53 R248Q cells and increased the number of cells with fragmented nuclei, consistent with replication stress-induced mitotic catastrophe in p53-deficient cells^25^ (Extended Data Fig. 5d, e). To test whether Cas12a2-damaged cells would be depleted from a heterogenous population, we performed a competitive growth assay in which p53 R248Q cells (labelled in red) were mixed with WT cells (labelled in green). Treatment with R248Q gRNA3 depleted p53 R248Q cells, allowing WT cells to dominate the population, whereas ACTB gRNA7 eliminated both cells (Extended Data Fig. 5f–h).

To determine whether endogenous *TP53* mutations can be targeted, we tested PC9 NSCLC cells harboring an endogenous p53 R248Q mutation (~83 transcripts per cell) in addition to EGFR E746_A750del (Extended Data Fig. 3h, i; 4f). In Cas12a2-expressing PC9 cells, transfection of R248Q gRNA3 caused strong growth inhibition and induction of DNA damage markers (Extended Data Fig. 5i, j). Remaining cells stained positive for cell-death markers after 96 h (Extended Data Fig. 5k,i), demonstrating efficient targeting of cancer cells carrying endogenous *TP53* point mutations.

### Cas12a2 distinguishes SNVs

**Extended data fig 6.**
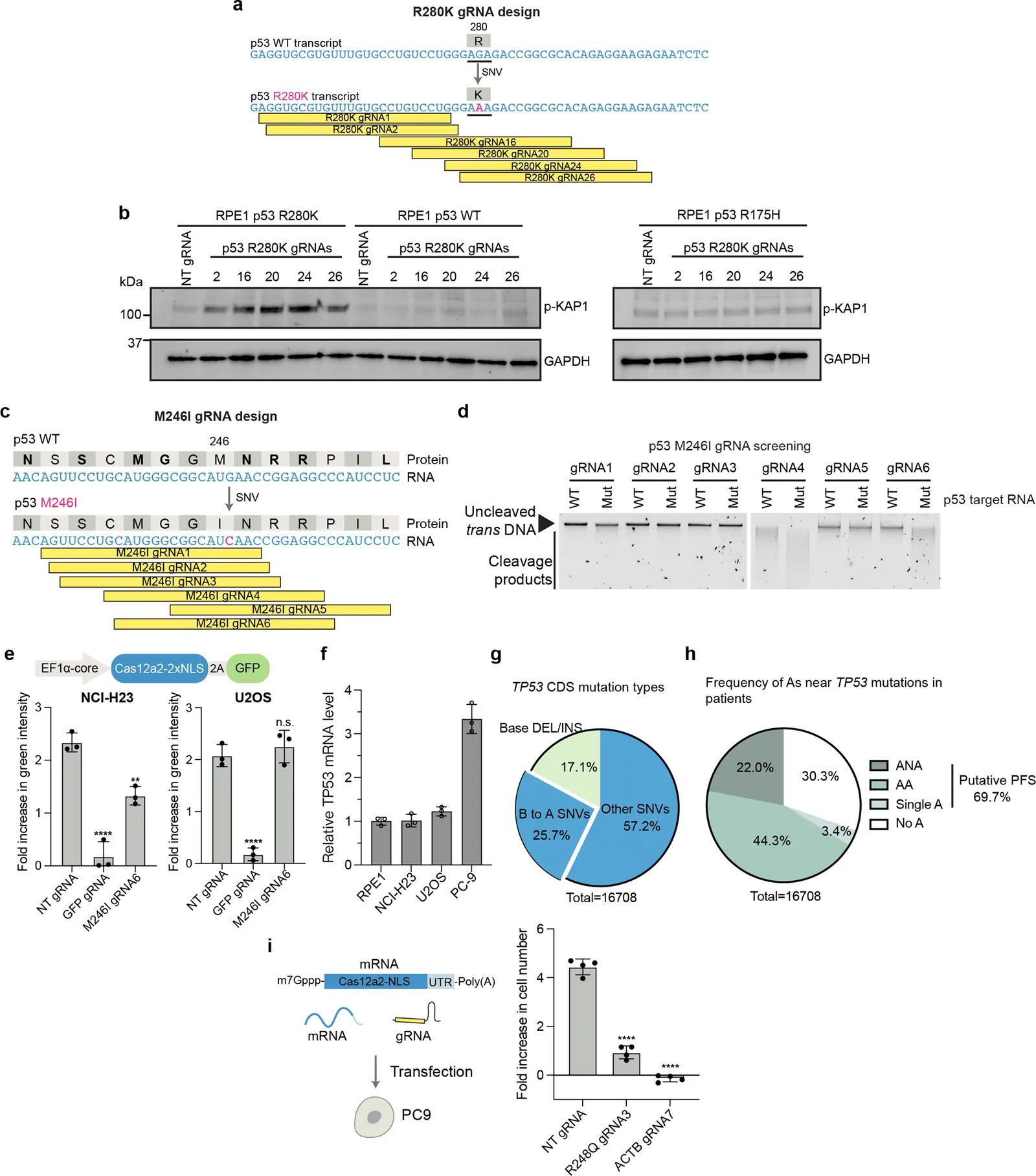
Cas12a2 shows selectivity when targeting mutations in the protospacer. **a,** Schematic showing annealing positions of R280K gRNAs that induced DNA damage in Extended Data Fig.4e. **b,** Immunoblots showing expression of DNA damage marker phospho-KAP1 24 h following Cas12a2 targeting of p53 R280K mutant transcript with 38.4 nM gRNA in target and control cells (n=3). **c,** Design of gRNAs targeting the p53 M246I mutant transcript. **d,** *Trans*-cleavage analysis of purified yeast genomic DNA by Cas12a2 with M246I gRNAs in the presence of the mutant target RNA or WT target RNA (n=3). **e**, Fold‑increase in green intensity over 96 h following Cas12a2 targeting of the p53 M246I mutant transcript in NCI-H23 cells (p53 M246I) and U2OS cells (p53 WT) expressing Cas12a2–2A-EGFP (n=3 independent experiments; mean ± SD is shown). Statistics: **p=0.0022; ****p<0.0001; n.s., non-significant; one-way ANOVA with Dunnett’s test**. f**. RT-qPCR analysis of *TP53* RNA levels in indicated cells (n=3 technical replicates; mean ± SEM is shown). **g,** Analysis of mutation types in the *TP53* coding sequence (CDS) from 16,708 tumor samples. Frequency of the presence of A, AA or ANA within 24 nt at the 3’ end of *TP53* mutations from 16, 708 tumor samples. Tumor sample data in **g** and **h** are from TCGA Pan-Cancer Atlas studies and MSK-CHORD studies. **i,** Cas12a2 targeting by transfecting mRNA encoding Cas12a2 and gRNAs into PC9 cells. Fold‑increase in cell number over 96 h is shown on the right (n=4 independent experiments; mean ± SD is shown). Statistics: ****p<0.0001, one-way ANOVA with Dunnett’s test.

We next examined whether selective targeting could also be achieved when the mutation was positioned within the spacer region. Among the six active R280K guides identified in the screen, four placed the mutation at different positions within the protospacer region (gRNA16, 20, 24, 26) (Extended Data Fig. 6a). These guides induced DNA damage in p53 R280K cells but not in WT or p53 R175H control cells (Extended Data Fig. 6b), indicating that a single mismatch in the protospacer reduces Cas12a2 activity.

We further tested spacer-based targeting using the p53 M246I mutation, which results from a G-to-C substitution in *TP53*. Cas12a2 showed *trans*-cleavage selectivity *in vitro* with one of the gRNAs (gRNA6) screened (Extended Data Fig. 6c, d). This gRNA induced growth defects in NCI-H23 cells harboring the endogenous M246I mutation but not in *TP53* wild-type U2OS cells with similar *TP53* expression levels (Extended Data Fig. 6e, f). Alongside the data presented above, these results demonstrate that in mammalian cells, Cas12a2 can discriminate between point mutations located either within the PFS or within the protospacer region of the target transcript.

To estimate the fraction of *TP53* mutations targetable using Cas12a2, we analyzed *TP53* coding sequence mutations in 16,708 patient samples to identify potential Cas12a2 target sites. We found 25.7% of *TP53* mutations in patients are B-to-A substitutions (where B is G, C, or T) (Extended Data Fig. 6g), which could serve as new or enhanced PFS for targeting. Additionally, for 69.7% of *TP53* mutations in patients, there were A, ABA or AA motifs within 24 nt at the 3’ end (Extended Data Fig. 6h), which could serve as a PFS to allow Cas12a2 targeting mismatches in the protospacer.

### Cas12a2 shows anti-tumor activity

Towards the goal of eventually using Cas12a2 RNPs in therapeutic applications, we tested whether Cas12a2 can be delivered as mRNA for cell killing. We generated capped and pseudo-uridylated mRNA encoding NLS-tagged Cas12a2 by *in vitro* transcription (IVT). Co-transfection of PC9 cells with this Cas12a2 mRNA along with p53 R248Q gRNA3 induced significant growth inhibition compared to controls transfected with non-targeting gRNA (Extended Data Fig. 6i).

**Extended data fig 7.**
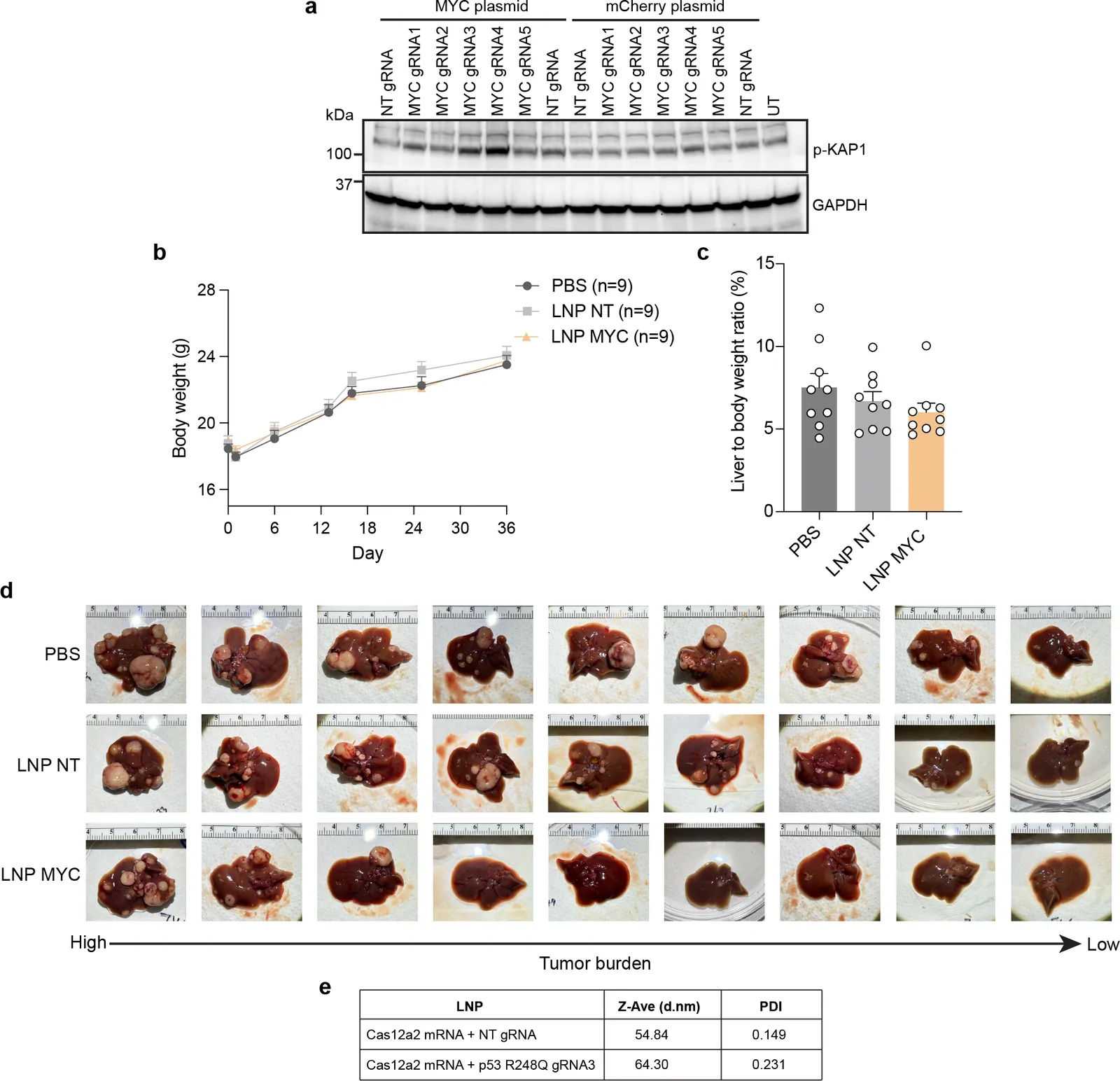
Targeting MYC with Cas12a2. **a,** HEK293 cells expressing Cas12a2 were transfected with a MYC-encoding plasmid or mCherry-encoding control plasmid. 24 hours later 38.4 nM gRNAs targeting MYC were transfected. Immunoblots probing expression of DNA damage marker phospho-KAP are shown (n=3). **b**, Changes in body weight of mice in Fig. 5c (n=9; mean ± SEM is shown). **c**, Quantification of liver to body weight ratios of mice in Fig. 5c at the endpoint (n=9; mean ± SEM is shown). **d,** Endpoint liver images of mice in Fig. 5c. **e,** Characterization of lung-enriching LNPs.

**Fig 5.**
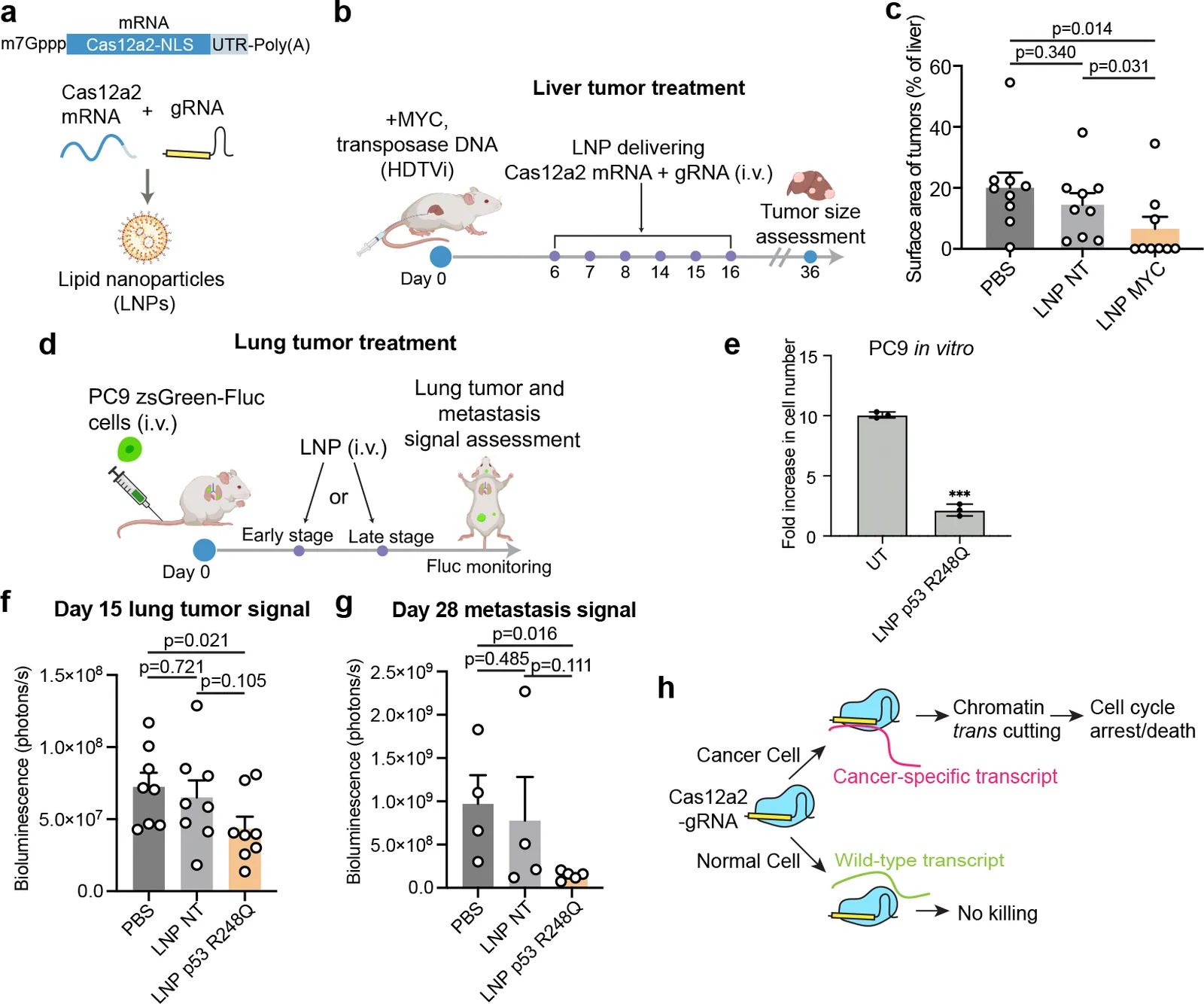
*In vivo* anti-tumor test. **a,** Schematic showing lipid nanoparticles (LNPs) packaging Cas12a2-encoding mRNA and gRNA. **b,** Schematic showing *in vivo* treatment of MYC-induced liver tumors. Plasmids expressing the MYC oncogene and Sleeping Beauty transposase were introduced into mice by hydrodynamic tail vein injection (HDTVi), resulting in stable integration of MYC into random liver cells. **c,** Quantification of liver tumor surface area as the percentage of total liver surface area (n=9; mean ± SEM). Statistical analysis was performed using a two-tailed Mann–Whitney U-test. **d,** Schematic showing *in vivo* treatment of PC9 lung tumors. Inoculated PC9 cells express firefly luciferase (Fluc) for live animal imaging. **e**, Fold‑increase in PC9 cell number over 96 h after LNP treatment *in vitro* (n=3; mean ± SD). ***p=0.0002, two-tailed unpaired t-test. **f**, Quantification of lung tumor bioluminescence at day 15 in mice from the early-stage treatment cohort (n=8; mean ± SEM). **g**, Quantification of bioluminescence signals at metastatic sites at day 28 in mice from the late-stage treatment cohort (n=4 or 5; mean ± SEM). Statistical analysis in **f** and **g** was performed using a two-tailed Mann–Whitney U-test. **h,** Schematic showing Cas12a2-mediated selective cancer cell elimination.

To evaluate the therapeutic efficacy of Cas12a2 *in vivo*, we first used a previously described MYC-induced liver tumor model^45^. In this model, the human *c-MYC* oncogene was stably integrated into random liver cells of tumor-prone FVB/NJ mice by transposases, resulting in liver tumor formation. We first screened gRNAs targeting MYC transcripts in Cas12a2-expressing HEK293 cells and identified one that induced strong DNA damage marker expression upon co-transfection with the MYC-expressing plasmid (Extended Data Fig. 7a). This gRNA (MYC gRNA4) was subsequently co-packaged with Cas12a2 mRNA into lipid nanoparticles (LNPs) for *in vivo* anti-tumor evaluation (Fig. 5a–c). Treatment began on day 6 post-tumor induction. Mice receiving MYC-targeting LNPs exhibited reduced tumor surface area (percentage of total liver) compared to control groups (Fig. 5c; Extended Data Fig. 7b–d).

**Extended data fig 8.**
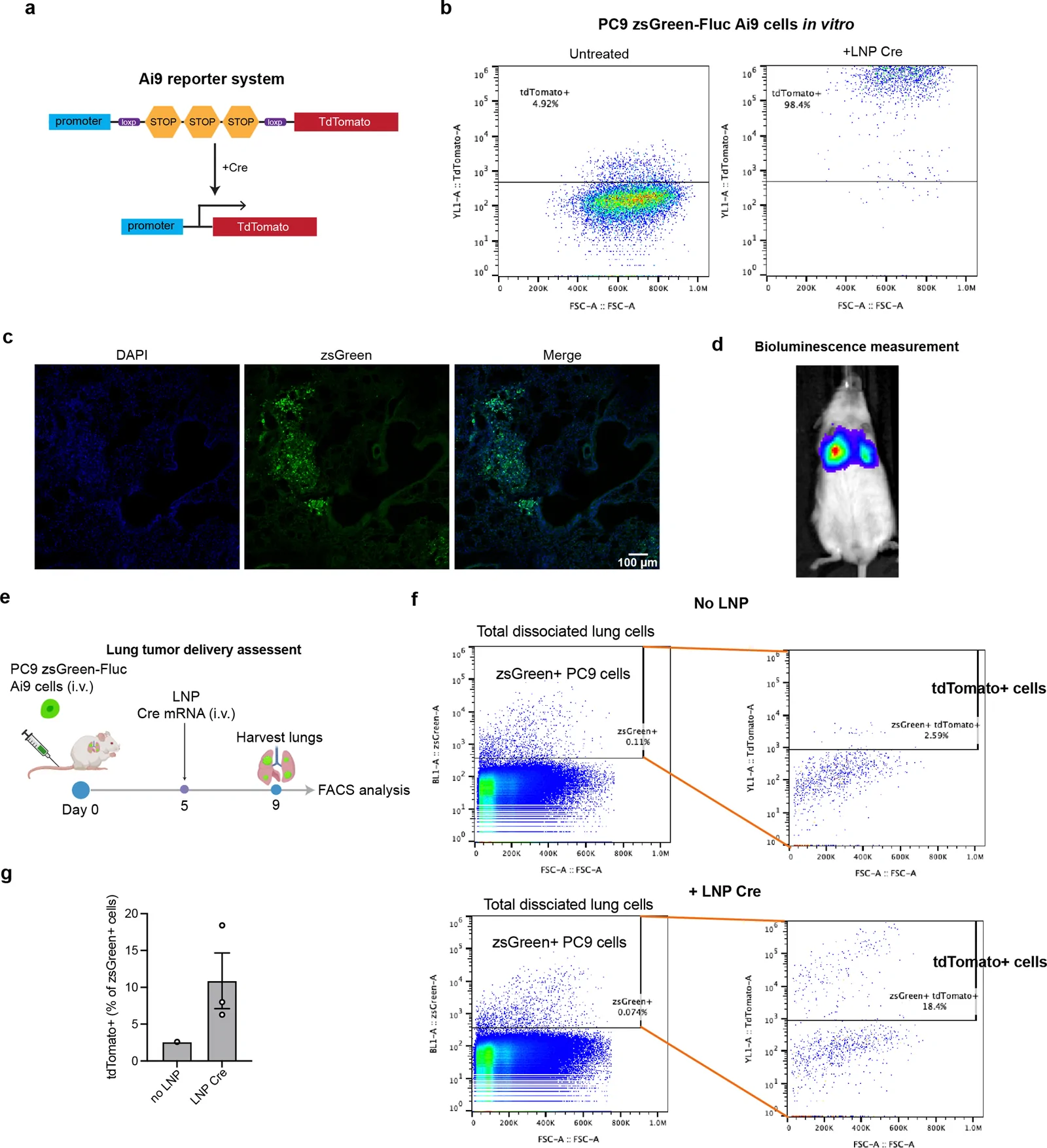
Lipid nanoparticles deliver mRNA to PC9 lung tumors in vivo. **a,** Schematic showing the Ai9 Cre-dependent tdTomato reporter system. The reporter construct was integrated into PC9 cells by the Sleeping Beauty transposase. Cells were selected by puromycin. **b,** Flow cytometry analysis showing tdTomato expression in PC9 Ai9 cells after treating them with LNPs delivering Cre mRNA *in vitro*. **c,** Fluorescent histological images (n=3) of a mouse lung 53 days post engraftment with PC9 zsGreen-Fluc cells. **d,** IVIS image of a mouse injected with PC9 zsGreen-Fluc cells. **e,** Schematic illustrating delivery efficiency assessment of LNPs to PC9 lung tumors. Each mouse received 20 μg Cre mRNA delivered by LNP. **f and g,** Flow cytometry analysis of isolated PC9 zsGreen Ai9 cells from mouse lung tissues after LNP Cre mRNA delivery as shown in **e.** Quantification of the percentage of tdTomato+ cells is shown in **g** (n=3 mice for LNP Cre treated group; mean ± SEM is shown).

Next, we evaluated p53 mutation targeting in mice bearing lung tumors (Fig. 5d). Lung-enriching LNPs have been previously developed^46–48^ via the SORT mechanism, with demonstrated delivery efficiencies of 15–20% for Cas9 editors in healthy lung tissues. In addition, SORT LNPs have also been able to deliver mRNA to lung metastases generated from A549 cells in mice^49^. We co-packaged Cas12a2 mRNA and p53 R248Q gRNA3 into lung-enriching LNPs^47^. Addition of these LNPs to cultured PC9 lung cancer cells induced significant growth inhibition (Fig. 5e; Extended Data Fig. 7e). We then assessed whether these LNPs could be used to deliver cargo to lung tumors *in vivo* using PC9 cells expressing zsGreen and a Cre-dependent tdTomato reporter (Extended Data Fig. 8a, b). Intravenous injection of PC9 cells established lung xenografts (Extended Data Fig. 8c, d). Following intravenous injection of a single dose of Cre mRNA-containing LNPs, ~7–18% of zsGreen-labeled PC9 cells turned on tdTomato expression within the lung xenograft (Extended Data Fig. 8e–g). Although this delivery efficiency was modest, we proceeded with anti-tumor testing using multiple dosing to compensate for the limited cellular uptake.

**Extended data fig 9.**
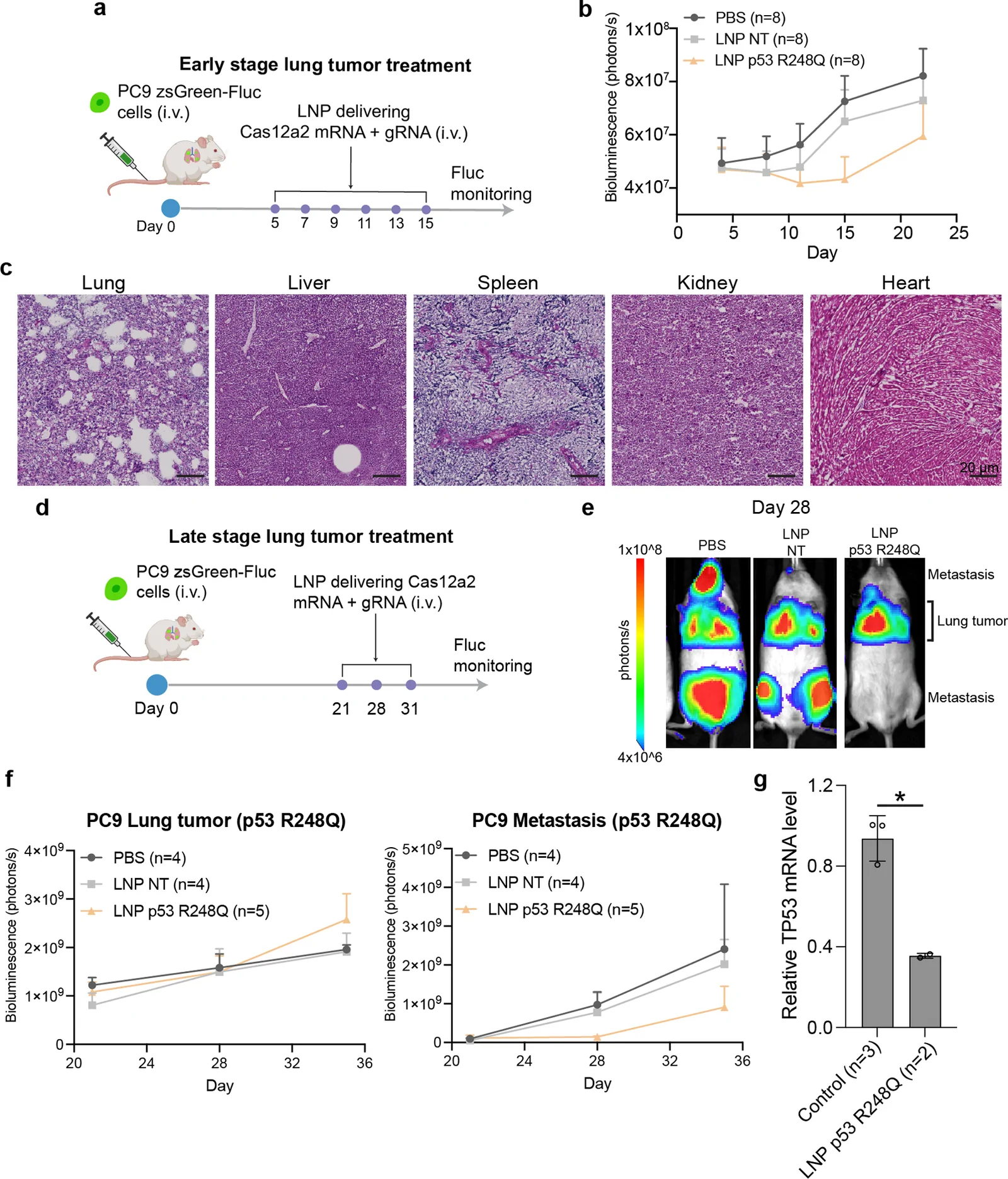
Cas12a2 treatment suppresses PC9 lung tumor progression in vivo. **a,** Schematic showing early-stage lung tumor treatment test. **b,** Tumor bioluminescence signals in the lung over time in treated mice in **a**. (n=8; mean ± SEM is shown). Statistical analysis of day 15 signals is shown in Fig. 5f. **c**, Representative histological images of mouse tissues from **a** (n=8 for lungs in each group; n=2 for other organs from LNP-treated groups). **d,** Schematic showing late-stage lung tumor treatment test. **e,** Representative IVIS images of mice bearing Fluc-expressing PC9 cells. Bioluminescence signals outside the lung are considered as tumor metastatic sites. **f,** Tumor bioluminescence signals in the lung and outside the lung over time in treated mice in **d**. (n is indicated in the figure; mean ± SEM is shown). Statistical analysis of day 28 signals is shown in Fig. 5g. **g**, RT-qPCR analysis of *TP53* RNA levels in PC9 cells recovered from treated mouse lungs (n=3; mean ± SEM is shown). *p<0.05, two-tailed unpaired t-test.

We engrafted 200,000 firefly luciferase (Fluc)-expressing PC9 cells into immunodeficient mice and initiated treatment 5 days later with six doses of LNPs co-delivering Cas12a2 mRNA and gRNA (Extended Data Fig. 9a). Tumor burden was monitored by bioluminescence imaging. Non-targeting LNPs showed a slight but non-significant decrease in tumor signal relative to PBS-treated controls (Fig. 5f; Extended Data Fig. 9b). A similar effect of SORT LNPs has been reported previously in lung tumor treatment^49^, suggesting that the LNP formulation itself may have a mild impact on tumor growth. In contrast, p53 R248Q-targeting LNPs produced a larger reduction in tumor signal that was statistically significant relative to PBS-treated animals. In addition, we observed no obvious tissue damage based on hematoxylin and eosin (H&E) staining of mouse lungs or other organs (Extended Data Fig. 9c), consistent with previous studies using the same LNP formulation^47,50^. To assess therapeutic potential in a more challenging context, we established an advanced-stage tumor model by engrafting mice with 1 million PC9 cells and allowing tumors to grow for 21 days prior to treatment initiation (Extended Data Fig. 9d). At day 21, luciferase signals in the lung approached saturation levels (Extended Data Fig. 9e, f). In this advanced setting, p53 R248Q-targeting LNPs did not reduce luciferase signal in the lung, but treatment was associated with delayed metastasis formation compared to PBS-treated controls (Fig. 5g; Extended Data Fig. 9f). In addition, tumors recovered from mouse lungs at the experimental endpoint showed significantly reduced *TP53* expression level in animals treated with p53 R248Q-targeting LNPs, suggesting that downregulation of the target transcript may represent a potential mechanism of resistance (Extended Data Fig. 9g). Taken together, these results suggest that nucleic acids encoding Cas12a2 and suitable gRNAs could be co-delivered to target cancer-specific transcripts for tumor suppression.

## Discussion

The results reported here establish Cas12a2 RNP-mediated chromatin shredding as an effective approach to selectively target cancer cells (Fig. 5h). We show that Cas12a2 cleaves eukaryotic chromatin *in trans* upon recognizing specific mRNA transcripts, triggering DNA damage responses and cell death in mammalian cells. This mechanism enabled us to target cancer cells with elevated *CCNE1* or *MYC* oncogene expression, *EGFR* in-frame deletion mutations and several *TP53* point mutations with high specificity.

The significance of these findings for cancer treatment could be considerable, particularly for cancers with undruggable mutations such as *TP53* mutations. Instead of losing TP53 function through genetic deletion, cancer cells more commonly preserve clonal mutant copies (Extended Data Fig. 4a) that confer selective advantages during tumor evolution^10,11,40,51–53^. Consequently, nearly all tumor cells driven by *TP53* mutations retain mutant *TP53* transcript expression. By targeting these ubiquitous mutant transcripts, Cas12a2 could overcome tumor heterogeneity. However, downregulation of the target transcript could represent a potential resistance mechanism to this approach. This could be addressed by multiplexed targeting of multiple cancer-associated transcripts. Cas12a2 can process its own CRISPR array^3^, potentially enabling simultaneous targeting of multiple transcripts from a single delivery. Additionally, targeting both the mutation and overexpression of the same transcript, as seen with overexpression of mutant MYC in certain cancer cases^54^, could provide a dual layer of efficacy and selectivity.

The design rules for effective Cas12a2 guides remain incompletely defined. We observed that the PFS requirements and mismatch discrimination in mammalian cells may differ from those characterized in bacterial systems, potentially reflecting differences in intracellular conditions such as Mg^2+^ concentration, or other factors. Guide RNA screens will need to be performed in human cells to establish generalizable Cas12a2 guide design principles, including the roles of PFS context, local RNA secondary structure, and spacer sequence composition. Further improvements in therapeutic efficacy will also require optimization of delivery systems, multiplexed targeting strategies, and engineering of Cas12a2 variants with enhanced *trans*-cleavage activity. Advances in these areas could substantially broaden the therapeutic potential of Cas12a2-based approaches.

No approved methods exist to directly target *TP53* mutations, leaving a critical gap given *TP53*’s importance in cancer. As the first approach to precisely target specific *TP53* mutations, our work paves the way for a new class of precision therapies using RNA-guided CRISPR nucleases.

## Methods

### Cell culture conditions.

HEK293, RPE1 and U2OS cell lines were cultured in DMEM (Corning) supplemented with 10% fetal bovine serum at 37 °C and 5% CO_2_. PC9 cells were cultured in RPMI1640 (Gibco) supplemented with 10% fetal bovine serum and 4mM total Glutamine at 37 °C and 5% CO_2_. Culture media were also supplemented with 1% penicillin/streptomycin.

### Cell lines.

PC9 cells were obtained from Sigma (90071810). RPE1 p21-GFP, RPE1 p53 R248Q FUCCI, RPE1 p53 R175H FUCCI cells were kindly gifted by John Diffley. U2OS TetON CCNE1 was previously described in Zeng et al., (2023)^25^ and was kindly gifted by John Diffley. All cell lines generated from this study are listed in Supplementary Table 4. Plasmid sequences used to generate stable cell lines used in this study can be found on Addgene and in Supplementary Table 4. Cell lines were not tested for mycoplasma contamination during the course of this study. No overt signs of contamination were observed during routine cell culture.

Parental hTERT-RPE1 cells had puromycin resistance due to the presence of a puromycin resistance gene (PuroR) on the hTERT plasmid used for cell line immortalization. PuroR was knocked out in RPE1 cells using CRISPR-Cas9 with a gRNA sequence 5’ GCAACCTCCCCTTCTACGAG 3’. RPE1 single cell colonies were selected and loss of puromycin resistance was validated with 0.5 μg/ml puromycin. These RPE1 cells without PuroR were used for downstream cell line generation. RPE1 p21-GFP was generated with CRISPR-Cas9 knock-in as previously described^55^. For generating RPE1 EGFR and p53 mutant cell lines, pLX313 plasmids carrying mutant *TP53* coding sequences and a Neomycin resistance gene (gifts from John Diffley) were used to make lentiviruses to transduce RPE1 cells. Stable cell lines were selected using 800 μg/ml G418. For stably expressing SuCas12a2 in cells, plasmids carrying the SuCas12a2 coding sequence, tagged with a nucleoplasmin NLS at the N-terminus, followed by P2A-PuroR (pJZ012 plasmid) or P2A-TagBFP (pJZ015 plasmid), were used to make lentiviruses as previously described^56^. After lentiviral transduction, cells stably expressing SuCas12a2 were selected using 2 μg/ml puromycin or confirmed with TagBFP expression.

PC9 cells expressing zsGreen-2A-Fluc (Firefly luciferase) were generated by lentiviral transduction. To introduce the Ai9 Cre-dependent tdTomato expression reporter into cells, we made a Ai9 donor plasmid (pZC007) suitable for genome integration by adding inverted terminal repeats (ITRs) flanking the Ai9 expression cassette and puromycin resistance gene. The donor plasmid pZC007 was then co-transfected with a Sleeping Beauty transposase-expressing plasmid pPGK-SB13 (Gift from Narita lab, Addgene 236078) into cells by lipofectamine 3000. Stable PC9 Ai9 cells were selected with 0.5 μg/ml puromycin.

For generating HEKGFP High, Mid and Low cells, single cell colonies were selected after transducing HEK293 cells with lentiviruses to express EGFP under a CMV promoter.

### Nucleic acid *trans*-cleavage assays.

Reaction mixes contained 250 nM SuCas12a2, 300 nM gRNA, 250 nM target ssRNA, and 100 nM FAM-labeled *trans* DNA or RNA substrate in 1x NEB3.1 buffer. For evaluating the effect of Mg^2+^ concentration, SuCas12a2, gRNA, and target ssRNA were pre-incubated at 37°C for 10 minutes in 1× NEB3.1 buffer (without MgCl_2_), then combined with the FAM-labeled *trans* DNA substrate and different concentrations of MgCl_2_ to initiate cleavage. Reactions were quenched at indicated timepoints by rigorous mixing with 10 μl phenol-chloroform; 4 μl of the aqueous layer after spinning was mixed with 4 μl 50% glycerol and resolved on 12.5% acrylamide Urea-Page denaturing gels, with cleavage products visualized on a Bio-Rad imager using the fluorescein channel. Substrate and target sequences are listed in Supplementary Table 3. Cleavage rates were calculated by fitting data to a one-phage association exponential model in GraphPad Prism.

### Chromatin *trans*-cleavage assays.

The assembled chromatin^57^ was made with yeast histones using a 10,577 bp plasmid (pGCL42), containing 8,645 bp yeast sequence surrounding ARS1, kindly provided by Giselle Lee and John Diffley. 50 μg of purified histone octamers was mixed with 50 μg of pGCL42 to a final volume of 200 μL in buffer A (25 mM HEPES-KOH pH 7.6, 1 mM EDTA) containing 1 M NaCl. The histone-DNA mix was loaded into a D-Tube^™^ Dialyser Mini (Merck Millipore) and dialysed at 4°C against 0.5 L buffer A of decreasing salt concentrations (1 M NaCl for 3 h, 0.75 M NaCl overnight, 0.5 M NaCl for 5 h, 0.0025 M NaCl overnight). After the final dialysis step, the dialysate is applied to a 5 ml 10%–40% v/v glycerol gradient (buffer A containing 0.0025 M NaCl) in a 13 × 51 mm tube (Beckman Coulter). Peak fractions containing reconstituted chromatin were pooled and assessed by micrococcal nuclease digestion. Chromatin was dialyzed against a storage buffer (25 mM HEPES-KOH pH 7.6, 2.5 mM NaCl, 0.1 mM EDTA) and stored at 4°C.

Chromatin cleavage reactions were performed at 37°C in 1× NEB3.1 buffer. The mix contained 14 nM SuCas12a2, 14 nM gRNA, 25 nM target ssRNA, and with 1 nM of a naked DNA plasmid or the assembled chromatin. Reactions were initiated by pre-incubating SuCas12a2, crRNA, and target ssRNA at 37°C for 15 minutes to form the RNP-target complex, followed by the addition of either plasmid DNA or chromatin in a master stock. 10 μl reactions were taken out and quenched with 10 μl phenol-chloroform at indicated time points. The aqueous layer of quenched reactions was taken out and mixed with an equal volume of 50% glycerol before being analyzed on 1% agarose gels. For MNase control reactions, 0.04 U MNase was added to either 1 nM of the naked DNA plasmid or the assembled chromatin in 1× NEB3.1 buffer supplemented with 5 mM CaCl_2_ at 37 °C.

To assess Cas12a2’s ability to degrade mammalian chromatin, 200,000 HEK293T cells were harvested, washed once with PBS, and lysed in 100 μl CSK buffer (10 mM HEPES-KOH pH 7.9, 400 mM NaCl, 1 mM MgCl_2_, 0.2% Triton X-100, 1 mM DTT, 1× protease-phosphatase inhibitors, 100 μg/ml BSA) at 4°C for 10 minutes. Then another 100 μl CSK buffer without NaCl was added to the lysed cells. Pre-incubated RNP-target RNA complex were prepared by incubating 250 nM SuCas12a2, 300 nM targeting gRNA or non-targeting gRNA, and 500 nM target ssRNA at 37°C for 10 minutes in 1× NEB3.1 buffer in 20 μl before addition to 200 μl nuclear extract (final concentrations: 250 nM SuCas12a2, 300 nM gRNA, 500 nM ssRNA). Reactions were incubated at 37°C, with aliquots taken at indicated time points, quenched with NTI buffer (from Takara NucleoSpin Gel and PCR Clean-Up Kit, 740609), and purified using the Takara kit, eluted in 15 μl elution buffer. Purified reaction products were visualized on 1% agarose gels.

### Cas12a2 targeting in mammalian cells.

For cells stably expressing Cas12a2, cells were seeded at ~5% confluence (~25,000 for RPE1, ~20,000 for PC9, ~40,000 for HEK293, ~30,000 for U2OS) in 1 ml media (DMEM + 10% FBS for RPE1, HEK293 and U2OS; RPMI1640 + 5% FBS + 2mM glutamine for PC9) in 24-well plates and reverse transfected with gRNAs. Transfection mixtures were prepared by combining Mix A (2 μl Lipofectamine RNAiMAX, 50 μl Opti-MEM) with Mix B (gRNA with indicated quantities, 50 μl Opti-MEM), incubating for 30 min, before adding to each well. For co-transfecting Cas12a2 mRNA and gRNA, transfection mixtures were prepared by combining Mix A (1.5 μl Lipofectamine MessengerMAX, 50 μl Opti-MEM) with Mix B (150 ng Cas12a2 mRNA, 130 ng gRNA, 50 μl Opti-MEM), incubating for 30 min, before adding to each well. For LNP delivery (see LNP formulation below), 150 ng mRNA and 150 ng gRNA co-packaged in LNPs were added to each 24-well. Incucyte (Sartorius) live-cell imaging were started 4 h post-seeding to monitor cell growth. All gRNA sequences used in this study are listed in Supplementary Table 1.

For nucleofecting Cas12a2 RNP into HEK293 cells, Cas12a2 RNP complex was prepared by combining Cas12a2 and gRNA (1:1 molar ratio) in 1× PBS (final RNP volume 5 μl) and incubated at room temperature for 10 minutes. 5.0 × 10⁵ HEK293 cells were centrifuged at 250 × g for 3 minutes, washed with 1× PBS, and resuspended in 20 μl nucleofection mix (16.4 μl SF Cell Line Solution, 3.6 μl Supplement 1; Lonza SF Cell Line 4D-Nucleofector X Kit) per condition before adding 5 μl RNP. 25 μl of the mixture was then transferred to a 16-well cuvette, nucleofected using the Lonza 4D-Nucleofector (program CA-189). Cells were then resuspended and seeded in 2 ml media in 6-wells. Incucyte (Sartorius) live-cell imaging was started 4 h post-seeding to monitor cell growth.

### Live cell imaging analysis.

Incutyte built-in AI confluence analysis and fluorescence analysis were used to measure cell confluence and GFP signal. For counting cell numbers, phase contrast images exported from Incucyte were segmented after Ilastik pixel classification training to identify individual nuclei. Segmented images were then analyzed using FIJI ImageJ to count cell numbers. GR50 (50% growth-rate inhibition concentration) was calculated as follows. Frist, cell number versus time data from logarithmic growth phase were fit by linear regression to obtain growth-rate slopes. Slopes were then normalized to the NT gRNA control. Normalized slopes were subsequently fit in GraphPad Prism using a dose-response model ([inhibitor] vs. response, variable slope), with the maximum constrained to 100.

### *In vitro* transcription (IVT) and gRNA.

Cas12a2 IVT DNA templates were amplified via Polymerase Chain Reaction (PCR) using Q5 High-Fidelity DNA Polymerase (New England Biolabs) and purified by treatment with 0.8 U Proteinase K and 1/5 volume of 10% SDS at 37 °C for 1 h, followed by heat inactivation at 95 °C for 10 min. DNA was extracted by 1 volume of phenol–chloroform–isoamyl alcohol. After centrifugation at maximum speed, the aqueous phase was recovered, mixed with 1/10 volume of 3 M sodium acetate (pH 5.2) and 2 volumes of cold 100% ethanol, and precipitated at −20 °C overnight. DNA was pelleted by centrifugation (≥10 min, 4 °C), washed twice with ice-cold 70% ethanol, air-dried, and resuspended in 20–100 μl RNase-free water. SUPER RNase inhibitor (1 μl; Thermo Fisher Scientific) was added to each sample. All steps were carried out in an RNase-free PCR workstation.

Cas12a2 mRNA was synthesized using the HiScribe T7 High Yield RNA Synthesis Kit (New England Biolabs) with IVT DNA templates described above. Reactions were mixed at room temperature with ATP, GTP, CTP and pseudouridine-5′-triphosphate included at equimolar concentrations (10 mM final concentration each). Co-transcriptional capping was performed with CleanCap AG (TriLink, 10 mM final). Reactions were incubated at 37 °C for 4 h, followed by treatment with 1 μl Turbo DNase (Thermo Fisher Scientific) at 37 °C for 15 min. RNA was then purified by lithium chloride precipitation. The reaction mix was mixed with 0.5 volumes of 7.5 M LiCl and incubated at −20 °C for at least 1 h. Precipitated RNA was collected by centrifugation (15–30 min, 4 °C), washed with ice-cold 70% ethanol, centrifuged, and resuspended in RNase-free water. RNA concentration was measured by Nanodrop and RNA integrity was assessed using denaturing formaldehyde agarose gels.

gRNAs were synthesized by Integrated DNA Technologies (IDT). All gRNA sequences used in this study are listed in Supplementary Table 1.

### Development of *in vivo* liver tumor models.

MYC liver tumor models were generated following previously published protocols^45,58^. Each female FVB/NJ mouse (Jackson Laboratory, Strain no. 001800) at 6–8 weeks of age was injected with 2 ml sterile PBS containing 10 μg pT3-EF1a-cMYC (Gift from Chen lab, Addgene 92046), expressing human MYC, and 1 μg pPGK-SB13 (Gift from Narita lab, Addgene 236078) plasmids via hydrodynamic tail vein injection (HDTVi) in 3 seconds. Animals were then randomized into treatment groups. All animal handling, care, treatment and euthanasia procedures were performed in accordance with guidelines established by the relevant Institutional Animal Care and Use Committee (IACUC). Body and liver weights were measured at the time of sacrifice to calculate liver-to-body weight ratios. Tumor nodules and whole liver were annotated manually as ROIs by FIJI, and areas of ROIs were measured. The percentage of whole liver area covered by tumors is calculated as follows: (sum of areas covered by tumors)/(whole liver area). Sample sizes were selected based on prior experience with the animal model and experimental feasibility. Investigators were not blinded to treatment group allocation during animal experiments or data analysis.

### Development of *in vivo* lung tumor model.

PC9 cells were injected intravenously into the tail vein of NOD.Cg-*Prkdc*^*scid*^ *Il2rg*^*tm1Wjl*^/SzJ (NSG) mice (Jackson Laboratory, Strain no. 005557) at 8–12 weeks old in 0.1–0.2 ml PBS to generate orthotopic tumors of NSCLC. For bioluminescence measurement of tumor burden, mice were injected intraperitoneally with 150 mg/kg D-Luciferin 10 min before being imaged using an IVIS spectrum imager. Animals were randomized into treatment groups after tumor implantation, with group sizes matched for baseline tumor burden where applicable. Bioluminescence images were analyzed on Living Image software. Sample sizes were selected based on prior experience with the animal model and experimental feasibility. Investigators were not blinded to treatment group allocation during animal experiments or data analysis. For delivery efficiency assessment, lung tissues were harvested, dissociated into single cells by digestion with collagenase and passing through 40 μm filters, before analysis by flow cytometry. Animals here and above were maintained on a 12-hour light/12-hour dark cycle, with ambient temperature controlled at 20–24 °C and relative humidity at 40–60%. Food and water were provided ad libitum. All animal handling, care, treatment and euthanasia procedures were performed in accordance with guidelines established by UCSF Institutional Animal Care and Use Committee (IACUC).

For histopathological analysis, mouse tissues were paraffin embedded, sectioned, placed on glass slides, stained with hematoxylin and eosin (H&E), digitally scanned to create whole slide images (WSIs). WSIs were uploaded to the Histowiz cloud platform and evaluated by a senior board-certified pathologist. WSIs were reviewed in entirety. None or minimal toxic changes were detected in all tissues examined.

### RT-qPCR and RNA-seq.

For RT-qPCR, total RNA was isolated using the RNeasy Mini Kit (Qiagen, 74104). cDNA was synthesized from 500 ng of total RNA using PrimeScript^™^ RT Master Mix (Takara, RR036A) according to the manufacturer’s instructions. RT-qPCR was performed using iTaq Universal SYBR Green Supermix (Bio-Rad, 1725121) on a Bio-Rad CFX96 system with the primers listed in Supplementary Table 2. For RNA-seq, 200,000 cells were harvested and lysed in 50 μl DNA/RNA Shield solution (Zymo). Samples were then submitted to Plasmidsaurus for RNA sequencing and downstream analysis.

### Lipid nanoparticle formulation.

4A3-SC8 (Catalog no. HY-148559) and 1,2-Dioleoyl-3-dimethylammonium-propane (DODAP, Catalog no. HY-130751) were purchased from MedChemExpress. 1,2-dioleoyl-sn-glycero-3-phosphoethanolamine (DOPE, Catalog no. 850725) and 1,2-Dimyristoyl-rac-glycero-3-methylpolyoxyethylen (DMG-PEG_2_K, Catalog no.880151) were purchased from Avanti Polar Lipids. Cholesterol (Catalog no. C8667) was purchased from Sigma. 1,2-dioleoyl-3-trimethylammonium-propane (DOTAP, Catalog no. D6182) was purchased from Sigma. For liver tumor treatment experiments, three doses of 10% DOTAP SORT (Selective Organ Targeting) LNP and three doses of 20% DODAP SORT LNP were used and formulated based on previously published protocols^59^. For lung tumor treatment experiments, the 40% DOTAP SORT LNP composition was used. The molar ratios for 4A3-SC8, DOPE, cholesterol, DME-PEG and DOTAP in 10% DOTAP and 40% DOTAP LNPs are 14.3:14.3:28.5:2.9:40 and 21.5:21.4:42.8:4.3:10 respectively. The molar ratio for 4A3-SC8, DOPE, cholesterol, DME-PEG and DODAP in 20% DODAP is 19.1:19:38.1:3.8:20. The total lipid to RNA weight ratio is 40:1 for 10% DOTAP LNPs, and 20:1 for 20% DODAP LNPs and 40% DOTAP LNPs. Lipids were dissolved in ethanol and mixed by microfluidics (NanoAssemblr Ignite) with RNA diluted in 10 mM citrate buffer (pH 4.0) at a lipid-to-RNA volume ratio of 1:3. The RNA payload consisted of mRNA and guide RNA combined at a 1:1 mass ratio. LNPs were then dialyzed (PurA-Lyzer Midi Dialysis Kits, WMCO 3.5 kDa, Catalog no. PURX35100) over night before being concentrated by centrifugation (Amicon Centrifugal Filter 30 kDa) at 2,000 g. Dynamic light scattering (DLS) measurements of LNPs were performed using Benano 180 Zeta Pro (Suzhou Benano Nanotech Co., Ltd., Cat. No. DLS180-Pro). In the *in vivo* anti-tumor tests, LNPs were dosed at 2 mg total RNA per kg body weight dissolved in 0.1–0.2 ml PBS via the lateral tail vein.

### RNA Single Molecule Fluorescence in Situ Hybridization (smFISH).

Cells were plated on coverslips, fixed with 4% paraformaldehyde (PFA) in PBS at room temperature (RT) for 15 minutes, washed three times with 1× PBS, permeabilized with 0.5% Triton X-100 in PBS for 10 minutes, and washed again three times with 1× PBS. Cells were then incubated in 30% formamide wash buffer at RT for at least 5 minutes, followed by hybridization with 50 μl of 3× hybridization buffer (30% vol/vol formamide, 0.1% wt/vol yeast tRNA, 0.1% vol/vol murine RNase inhibitor, 10% vol/vol dextran sulfate in 2× SSC) containing RNA smFISH probes (Supplementary Table 2) at 37°C overnight. Samples were then washed twice with 30% formamide wash buffer at 37°C for 30 minutes each, followed by two washes with 2× SSC, and either stored in 2× SSC with RNase inhibitor or immediately processed for readout staining by incubating with 10% ethylene carbonate (EC) hybridization buffer (10% vol/vol EC in 2× SSC) containing 3 nM readout probe (5’-ATTO590-ATCCTCCTTCAATACATCCC-3’) at RT for 15 minutes, washing twice with 2× SSC, and imaging on a confocal microscope with 63x or 100x oil immersion lens.

### Antibodies.

Immunoblotting was performed using the following antibodies diluted in TBS buffer supplemented with 0.1% Tween 20 and 5% milk powder or 3% BSA: p-KAP1 (1:1000, Bethyl Laboratories, A300–767A), p-H2A.X S139 (1:1000, Millipore, 05–636), GAPDH (1:1000, Santa Cruz, sc-365062), anti-Mouse HRP(1:5000, Invitrogen, 31430), anti-Rabbit HRP (1:5000, Invitrogen, 65–6120), anti-Rabbit IRDye800 (1:5000, Licor, 926–32211) and anti-Mouse IRDye680 (1:5000, Licor, 926–68070).

## Extended Data

## Supplementary Material

supplementary table 1

supplementary table 3

supplementary table 2

supplementary table 4

supplementary table 5

Additional Information

Supplementary Information is available for this paper. Correspondence and requests for materials should be addressed to Jennifer A. Doudna. Reprints and permissions information is available at www.nature.com/reprints.

## Data Availability

RNA-seq data are publicly available at NCBI GEO under accession GSE332893. Gene mutation data from TCGA Pan-Cancer Altas and MSK-CHORD studies are available for public download at cbioportal.org. All gRNA, target, and plasmid sequences used in this study are provided in the Supplementary Information. Source data are provided.

## Code availability

Code and data used calculate the fraction of *TP53* mutations potentially compatible with SuCas12a2 targeting are available at https://github.com/zeng-j-k/TP53-mutation-compatibile-SuCas12a2-PFS.git. Python 3.13 was used to analyze the data.
